# Analysis of Marker-Defined HNSCC Subpopulations Reveals a Dynamic Regulation of Tumor Initiating Properties

**DOI:** 10.1371/journal.pone.0029974

**Published:** 2012-01-20

**Authors:** Paloma Bragado, Yeriel Estrada, Maria Soledad Sosa, Alvaro Avivar-Valderas, David Cannan, Eric Genden, Marita Teng, Aparna C. Ranganathan, Huei-Chi Wen, Avnish Kapoor, Emily Bernstein, Julio A. Aguirre-Ghiso

**Affiliations:** 1 Division of Hematology and Oncology, Department of Medicine, Mount Sinai School of Medicine, New York, New York, United States of America; 2 Department of Otolaryngology, Mount Sinai School of Medicine, New York, New York, United States of America; 3 Department of Oncological Sciences, Mount Sinai School of Medicine, New York, New York, United States of America; 4 Department of Dermatology, Mount Sinai School of Medicine, New York, New York, United States of America; 5 Tisch Cancer Institute, Mount Sinai School of Medicine, New York, New York, United States of America; 6 Black Family Stem Cell Institute, Mount Sinai School of Medicine, New York, New York, United States of America; Institute for Systems Biology, United States of America

## Abstract

Head and neck squamous carcinoma (HNSCC) tumors carry dismal long-term prognosis and the role of tumor initiating cells (TICs) in this cancer is unclear. We investigated in HNSCC xenografts whether specific tumor subpopulations contributed to tumor growth. We used a CFSE-based label retentions assay, CD49f (α6-integrin) surface levels and aldehyde dehydrogenase (ALDH) activity to profile HNSCC subpopulations. The tumorigenic potential of marker-positive and -negative subpopulations was tested in nude (Balb/c nu/nu) and NSG (NOD.Cg-*Prkdc^scid^ Il2rg^tm1Wjl^*/SzJ) mice and chicken embryo chorioallantoic membrane (CAM) assays. Here we identified in HEp3, SQ20b and FaDu HNSCC xenografts a subpopulation of G0/G1-arrested slow-cycling CD49f^high^/ALDH1A1^high^/H3K4/K27me3^low^ subpopulation (CD49f+) of tumor cells. A strikingly similar CD49f^high^/H3K27me3^low^ subpopulation is also present in primary human HNSCC tumors and metastases. While only sorted CD49f^high^/ALDH^high^, label retaining cells (LRC) proliferated immediately *in vivo*, with time the CD49f^low^/ALDH^low^, non-LRC (NLRC) tumor cell subpopulations were also able to regain tumorigenic capacity; this was linked to restoration of CD49f^high^/ALDH^high^, label retaining cells. In addition, CD49f is required for HEp3 cell tumorigenicity and to maintain low levels of H3K4/K27me3. CD49f+ cells also displayed reduced expression of the histone-lysine N-methyltransferase EZH2 and ERK1/2phosphorylation. This suggests that although transiently quiescent, their unique chromatin structure is poised for rapid transcriptional activation. CD49f− cells can “reprogram” and also achieve this state eventually. We propose that in HNSCC tumors, epigenetic mechanisms likely driven by CD49f signaling dynamically regulate HNSCC xenograft phenotypic heterogeneity. This allows multiple tumor cell subpopulations to drive tumor growth suggesting that their dynamic nature renders them a “moving target” and their eradication might require more persistent strategies.

## Introduction

Head and neck squamous cell carcinoma (HNSCC) is an aggressive disease for which treatment has improved little over the last three decades. A complicating factor is that despite the discovery of certain genetic alterations and an HPV+ subgroup of tumors, the genetics and cell biology of this cancer is heterogeneous and poorly characterized, while the epigenome remains an enigma [1], [2]. A critical, but only recently explored question is the existence and behavior of tumor subpopulations in HNSCC that may or may not behave like tumor initiating cells (TICs).

HNSCC primary tumors have been shown by Prince *et al.* to contain a subpopulation of CD44+/Lin− cells, which correlated with TIC potential [3]. CD44+ expression also correlated with higher expression of Bmi1, a polycomb group PRC1 complex component that controls self-renewal [4]. Recently this same group demonstrated that the CD44+ population overlaps with an aldehyde dehydrogenase ALDH1A1^high^ subpopulation that displayed enhanced tumorigenicity [5]. Hierarchical or non-hierarchical models are proposed to explain the behavior of marker-defined tumor cell subpopulations. The first, proposes that the TIC compartment is a distinct minority and that these cells have the ability to self-renew as well as produce identifiable progenitor, transit amplifying or differentiated cells [6], [7]. The non-hierarchical model proposes that TICs result from the random occurrence of TIC-specific or other (e.g., clonally selected) properties and any cell in the tumor can be a TIC [8], [9], [10], [11], [12]. The data on the existence and functionality of TICs for different human malignancies is conflicting. For example, in human melanoma marker defined subpopulations of tumor cells have been shown to be responsible for tumor initiation and growth [13], [14], [15]. However, other studies showed that tumor expansion in this malignancy (as well as in colon carcinoma or mouse leukemias) was propelled by genetically distinct clones or by operationally defined TICs that occurred at high frequency [8], [9], [11], [16]. Importantly, it is now clear that this apparent discrepancy between studies might arise from the fact that the results are highly dependent on the culture conditions, how tumors were enzymatically digested to produce single cell suspensions [8], [15] and the degree of immuno-deficiency of the xenograft model used [9], [13]. While there is strong support for the existence of molecularly-defined TICs in certain tumors, it is also possible that a dynamic regulation of “stem-cell properties” might explain models that seem to display a non-hierarchical behavior [17], [18]. This mechanism may provide transient populations with a TIC potential that dynamically and reversibly establish a hierarchical structure during tumor growth.

A growing number of experimental and theoretical studies suggest that the TIC phenotype might be more dynamic than expected [17], [18]. These studies motivated us to explore whether a dynamic behavior of discrete tumor cell subpopulations might drive HNSCC tumor growth. Studies in the HEp3 HNSCC model showed that *in vivo* serially transplanted tumors retain their tumorigenicity and display >90% engraftment in Balb/c nude mice or chick embryo systems (CAM) [19], [20]. Further, when these tumor cells were cloned at nearly 100% efficiency, so that primary tumor heterogeneity was well represented, all clones (10^2^), albeit after different latency periods, were invariably tumorigenic and transplantable [20]. These studies provided an initial hint that in HNSCC and perhaps other tumors, a dynamic regulation of tumor initiating properties drives tumorigenic potential.

Here we identify, in experimental models of HNSCC a tumor cell subpopulation defined as transiently G1 arrested, CD49f^high^/ALDH1A1^high^/P-ERK1/2^low^/H3K27/K4me3^low^ cells (CD49f+) with immediate enhanced engraftment capacity. This CD49f^high^/H3K27me3^low^ subpopulation was also found in specimens from human primary tumors and metastases. Importantly, our data revealed that these phenotypes are dynamic and even pure CD49f^low^ (CD49f−) subpopulations can eventually give rise to CD49f+ cells with tumorigenic potential. This associates with specific changes in ERK1/2 activation, histone H3 post-translational modifications and with changes in the expression of CD49f (α6-integrin) and ALDH activity. Thus, we identify at least two coexisting subpopulations in growing tumors that only differ in the timing of their tumorigenicity rather than in their overall potential. This work might reveal novel dynamics on how phenotipically heterogeneous subpopulations of tumor cells maintain solid tumor growth.

## Results

### ALDH1A1^High^/CD49f^High^ Tumor Cells Define a Slow-Dividing Subpopulation in HNSCC HEp3 tumors

**Figure 1:**
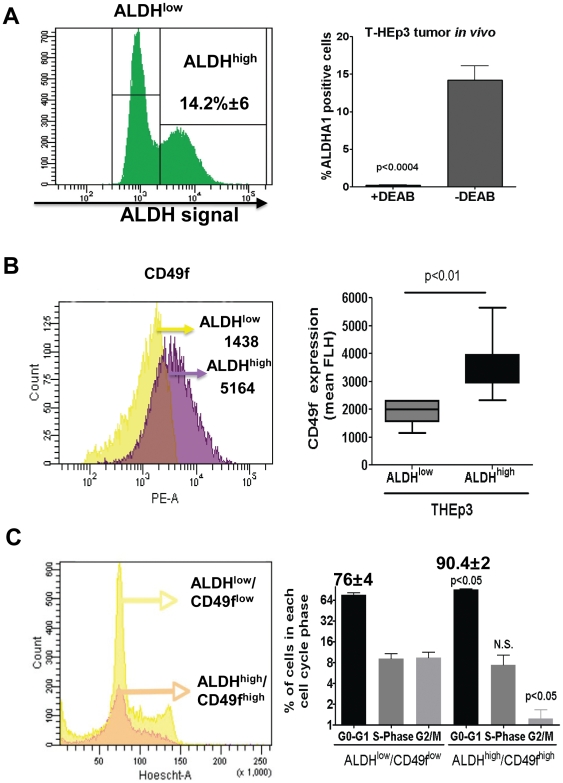
T-HEp3 Cells Contain an ALDH1A1^high^, CD49f^high^ and CD44^high^ Cell Sub-population. (**A**) FACS analysis of ALDH1A1 activity in T-HEp3 tumors grown in nude mice. Left- Representative Histogram of ALDH1A1 expression. Percentages = frequency of ALDH^high^ cells. Right- quantification of the percentage of ALDH1A1^high^ cells in at least 3 different HEp3 tumors. (**B**) FACS analysis of CD49f expression in the ALDH^high^ and ALDH^low^ subpopulations. Left - representative histograms, with numbers indicating the CD49f mean fluorescence intensity (MFI). Right - quantification of CD49f MFI in at least 3 different HEp3 tumors. (**C**) HEp3 tumor cells stained for ALDH1A1 activity, CD49f and Hoechst were FACS analyzed for cell cycle profile. Cell cycle profile (left) and quantification (right). Percentage of cells was calculated using BD FACSDiva software, excluding cell doublets and aggregates. Columns - mean of three independent experiments. The numbers represent the percentage of cells on G0-G1phase. p-values estimated using Mann-Whitney non-parametric test.

High aldehyde dehydrogenase-ALDH1A1 activity (henceforth ALDH) was shown to identify hematopoietic, breast and colon cancer stem cells [21], [22], [23]. We examined the distribution of ALDH activity in HEp3 cells obtained from tumors grown on chicken embryo chorioallantoic membrane (CAM) that provides a completely immune-deficient environment where these tumor cells grow for one week and can be weekly passaged [19], [20], [24] or nude mice according to published methods [23]. HEp3 tumorigenicity in nude mice and CAM system [19], [25], [26] as well as in NSG mice (this study) are virtually indistinguishable. This analysis revealed that while the majority of HEp3 cells had low ALDH activity, a subpopulation comprising ∼14% of the tumor is ALDH^high^ (14.20±6.05%, p = 0.00006) and this activity is sensitive to DEAB (ALDH inhibitor) (Fig. 1A). Detection of surface CD49f, a surface integrin (α6) upregulated in HNSCC tumors and also a marker of normal stem cells in various tissues [27], [28], [29], [30], showed that freshly harvested HEp3 cells from tumors or SQ20b and FaDu cells from culture expressed high levels of this integrin (Fig. S1A). Moreover, ALDH^high^ HEp3 cells obtained from CAM tumors express in average ∼2 fold higher levels of surface CD49f than ALDH^low^ HEp3 cells (3527±1116 vs. 1908±437, p = 0.008) (Fig. 1B) and in some tumors these differences reached a maximum of 4 fold (Fig. 1B). In agreement with previous studies [31], [32], we also found that ALDH^high^ HEp3 cells displayed slightly higher levels of surface CD44 when compared to ALDH^low^ HEp3 cells (1.3 folds higher, 1793±345 vs. 1331±233, p = 0.0026) (Fig. S1B). ALDH^high^ HEp3 cells were negative or showed no difference from the ALDH^low^ counterpart for other defined markers such as CD71 (6354±979 vs. 5307±1217, p = 0.52) (Fig. S1C), CD34 [33], [34], CD200 [33] and CD133 [35] (data not shown). We conclude that HNSCC HEp3 tumors contain a sub-population of ALDH^high^/CD49f^high^ cells.

TICs such as leukemic stem cells are proposed to retain stem cell-like properties, such as quiescence [36]. Therefore, we analyzed the cell cycle distribution of ALDH^high^/CD49f^high^ vs. ALDH^low^/CD49f^low^ cells in freshly harvested HEp3 tumors by combining detection of ALDH activity, CD49f expression and DNA content and viability using Hoechst (Fig. 1C – triple stain). In contrast to ALDH^low^/CD49f^low^ HEp3 cells that showed a cell cycle distribution consistent with active cycling (76±8% G0/G1, 18.36±5.9% S+G2/M), ALDH^high^/CD49f^high^ HEp3 cells were found mainly in G1 and very few cells were found in G2+M phases (90.4±3.9% G0/G1, 8.6±5.4% S+G2/M) (Fig. 1C). This suggests a slow cycling behavior for the ALDH^high^/CD49f^high^ cells. Consistent with previous results [37] the percentage of sub-G0 apoptotic cells was low, ranging 2–6% in HEp3 tumors (not shown). We conclude that ALDH^high^/CD49f^high^ cells are primarily in a G1-arrest, while ALDH^low^/CD49f^low^ cells in the tumor mass show a profile consistent with active expansion.

### Label Retaining Cells are Enriched for CD49f in HEp3 primary Tumors

**Figure 2:**
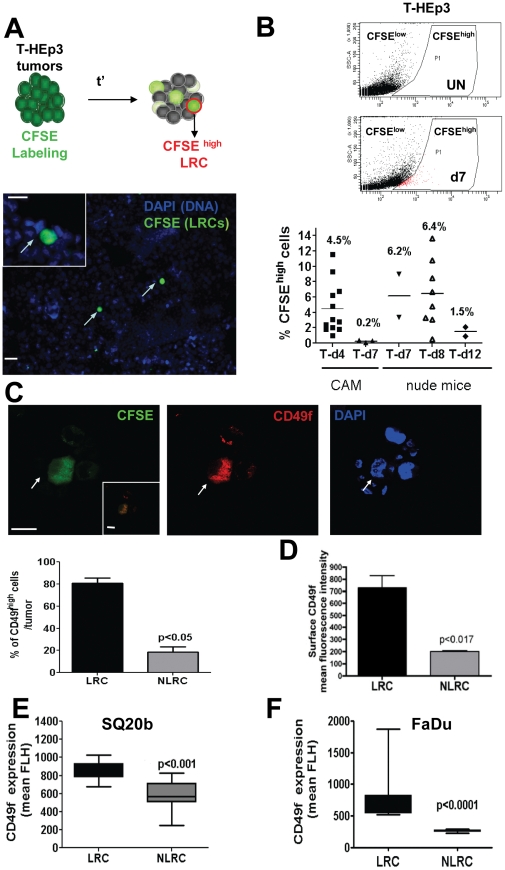
CD49f^high^ Cells Are A Slow Cycling Population in T-HEp3 Tumors. (**A**) Upper panel, strategy to use CFSE (20 µM) to identify LRCs in HEp3 tumors *in vivo*. CFSE diffuses into the cells and is esterified onto proteins. Cell division dilutes the label and the slow-cycling or growth arrested cells will retain the CFSE (label retaining cells - LRCs). Lower photograph - cytospin from a CFSE-labeled 4 day T-HEp3 tumor grown on the CAM. The LRCs (green cells, arrows) can be easily identified. Scale bar: 80 µm and 40 µm inset scale bar. (**B**) FACS detection and quantification of the CFSE population after *in vivo* growth. Upper panel - dot-plot graph of unstained (UN) T-HEp3 tumors; middle panel - CFSE labeled tumors after 7 days (d7) *in vivo*. Lower panel - percentage of LRCs determined by fluorescence microscopy in HEp3 (T) tumors grown on CAMs or nude mice d4–d12 after injection. Top numbers = mean percent of LRCs per tumor. (**C**) Detection of CD49f in LRCs and NLRCs in d6 CAM tumors by IF. LRCs (green) are positive for CD49f (red), arrows. Inset, merged image of CD49f and CFSE signals. CFSE signal bathes the whole cell but primarily the cytosol. CD49f signal in permeabilized HEp3 cells excluded completely the nucleus (Blue, DAPI). Scale bar: 60 µm and 40 µm inset scale bar. Lower panel graphs - quantification of CD49f^high^ cells in both LRC and NLRC populations. (**D**) Quantification of surface CD49f^high^ cells in both LRC and NLRC populations by FACS. p-values estimated using Mann-Whitney non-parametric test. (**E–F**) FACS quantification of CD49f expression in LRCs and NLRCs in SQ20b (**E**) and FaDu (**F**) tumors grown in nude mice. Statistical significance was estimated using Mann-Whitney non-parametric test and 95% confidence interval.

The G1 arrest observed in ALDH^high^/CD49f^high^ cells motivated us to use a functional approach to search for slow cycling cells using a label retaining assay using the vital green-fluorescent dye CFSE (Fig. 2A) [38]. CFSE label retention provides a functional assay for a phenotype associated with normal stem cell behavior (i.e. quiescence) and also it provided a functional and unbiased method for which we could then test overlap with the molecularly defined populations. T-HEp3 cells from freshly harvested tumors were loaded with CFSE (20 µM) for 20 min, washed and injected in nude mice or CAMs. CFSE had no effect on cell viability or proliferation rates (not shown). Detection of CFSE+ HEp3 cells in cytospins within one week of CAM tumor growth (day 4 shown) revealed a small fraction of LRCs (Fig. 2A lower panel). After collagenase-I mediated digestion of tumors and FACS analysis to detect the CFSE+ population we found that tumor cells displayed varying degrees of CFSE signal consistent with the number of divisions (Fig. 2A and Fig. S1D). FACS analysis revealed that label retaining cells (LRC) comprised 4% of the tumor obtained from CAMs (4 days). This proportion is further decreased by day 7 in CAM tumors (Fig. 2B) and it represents 6% of the tumor cell population in nude mice-grown HEp3 tumors for 7–8 days. This proportion also decreases by day 12 in nude mice (Fig. 2B). In fact, the CFSE-marked LRCs were no longer detectable after 2 weeks *in vivo* in either system CAM or nude mice, indicating that all LRCs divided enough times to completely dilute the label. However, if the cells derived from these tumors that had diluted the label were relabeled with CFSE and reinoculated *in vivo*, a LRC subpopulation was again obtained after one week of *in vivo* growth. This suggests that although all LRCs eventually dilute the label (i.e. cycle) this population is regenerated. Propidium iodide or Hoechst labeling indicated that CFSE retaining cells are viable (data not shown).

In order to determine whether the CD49f^high^ cells partitioned preferentially to the quiescent label retaining tumor cell population or whether these methods identified distinct populations, CFSE^high^ cells were used to detect CD49f surface expression through immunofluorescence (IF) and FACS. Detection of CD49f was performed in HEp3 LRCs and non-LRCs (NLRCs) obtained from 6 days old CAM tumors. As shown in figure 2C close to 80% of all HEp3 LRCs are CD49f^high^ (Fig. 2C and Fig. S1E). The CD49f signal was cytoplasmic and membranous because cells were permeabilized revealing integrins in the ER and Golgi. This method allows only scoring positive vs. negative cells using a threshold of signal detection by the microscope camera. To further quantify these differences we measured CD49f expression by FACS (surface CD49f) and found that HEp3 LRCs (CFSE+) express 3.5 fold more surface CD49f (mean fluorescence intensity) than NLRCs (Fig. 2D). This strongly suggests that the LRC/CD49f^high^ sub-population overlaps with the ALDH^high^/CD49f^high^ sub-population that shows the highest degree of CD49f expression (Fig. 1B, 2D).

CFSE labeling of SQ20b cells and injection in nude mice revealed after 7–14 days *in vivo* the presence of a slow-cycling LRC population that represented 1.5±0.7% of tumor cells (Fig. S1F). Quantification of CD49f expression by FACS in LRCs and NLRCs from SQ20b and FaDu tumors grown in nude mice showed that the LRCs population expressed statistically significant higher levels of CD49f (Fig. 2E and 2F) (SQ20b: 972.6±150.8 vs. 551.6±172.1, p<0.0001. FaDu: 1752.6±92.8 vs. 261.6±4.0, p<0.0001). Collectively, these data suggests that in tumors produced by at least three different HNSCC cell lines (one serially maintained *in vivo* – HEp3) a subpopulation of CD49f^high^ cells appears to be transiently growth arrested.

### A CD49f^high^ Sub-population is Present in Primary tumors and Metastasis from HNSCC patients

**Figure 3:**
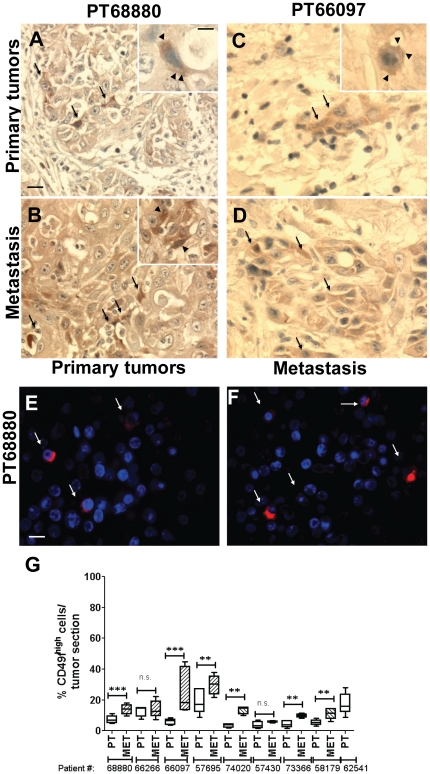
Primary tumors and metastasis from HNSCC patients contain a subpopulation of CD49f^high^ cells. (**A–D**) Representative CD49f staining in primary tumors (A and C) and lymph node metastasis (B and D) from two different patients. CD49f^high^ tumor cells are marked with arrows. Note the strong cytosolic and membrane pattern of the signal (arrowhead in the, inset) and the overall stronger signal and increased frequency for CD49f in the metastatic lesions (B and D). Scale bar: 80 µm and 40 µm inset scale bar. (**E–F**) Immunofluorescence detection of CD49f in sections from human oral primary tumors (E) and metastasis (F) (PT68880). Scale bar: 60 µm. (**G**) Quantification of CD49f^high^ cells in sections from primary tumors and lymph node metastasis. Columns represent mean of 5 different sections per sample and a minimum of 500 cells was scored per sample. p-values estimated using one-way ANOVA followed by the Bonferroni correction with two-tailed *P* values<0.05 considered significant.

CD49f has been identified as a normal stem cell marker and overall expression of CD49f is increased in human HNSCC tumors [27], [28], [30], [39], [40]. Thus, we tested in sections from human HNSCC primary tumors and metastasis whether a CD49f^high^ cell subpopulation was also present. Staining with the anti-CD49f antibody showed that CD49f expression was higher in the basal layer of cells of the adjacent mucosa (Fig. S2A). CD49f staining decreased in the suprabasal more differentiated cell layers. Examination of 9 primary tumors and 8 matching lymph node metastases using IHC or immunofluorescence (Fig. 3A–F) (representative of poorly differentiated squamous cell carcinoma with high sarcomatoid differentiation) revealed that CD49f expression in these tumors was limited to subpopulations representing 5–18% (Fig. 3) of all tumor cells. The very strongly stained CD49f cells that showed a characteristic membrane and cytoplasmic staining were interspersed in the tumor mass (Fig. 3). The proportion of this subpopulation was similar to that detected in HEp3 tumors using FACS, IF (Fig. 2) and immunohistochemistry (IHC) (Fig. S2B). The frequency of CD49f^high^ cells significantly increased in 75% (6/8) patient metastases (12–30%) (Fig. 3G and Fig. S2C) compared to primary lesions and this was accompanied by increased staining intensity (Fig. 3B and D). Thus, a CD49f^high^ cell subpopulation detected in HNSCC cell lines is present in aggressive tumors and enriched in lymph node metastases from HNSCC patients. This suggests that the CD49f^high^ population studied in the experimental systems might be informative of this CD49f^high^ population found in tumor samples obtained from patients.

### Both ALDH^High^/CD49f^High^ Cells and LRCs Display Enhanced Proliferative Potential *In Vivo*

**Figure 4:**
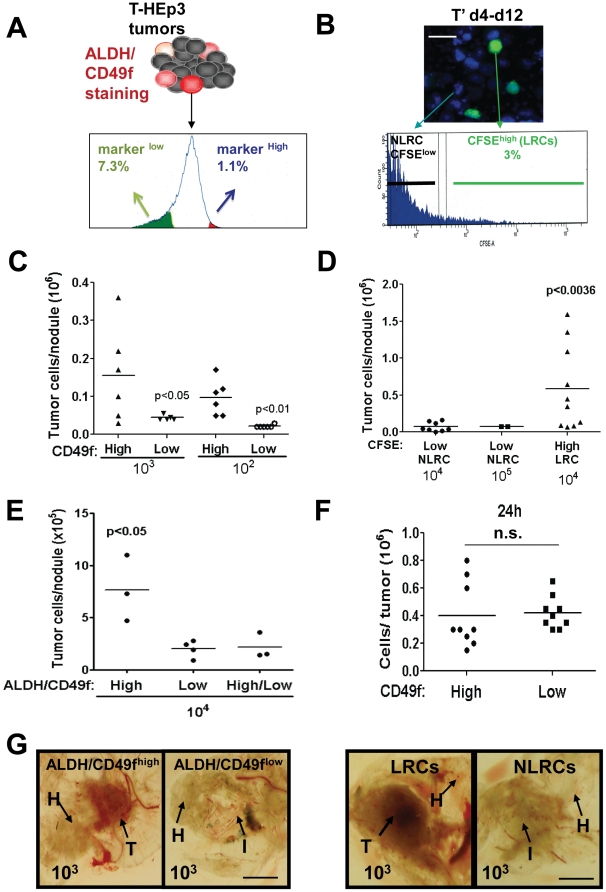
ALDH^high^, CD49f^high^, LRCs proliferative capacity *in vivo*. (**A**) Sorting strategy for HEp3 cells with high or low levels of ALDH, CD49f, or both. (**B**) Sorting strategy for HEp3 tumors labeled with CFSE. (**C–E**) Quantification of tumor growth after 1 week *in vivo* on CAM. Dots represent the number of tumor cells per nodule. CD49f^high^ vs. CD49f^low^ cells (**C**), LRC vs. NLRCs (**D**), and (**E**) ALDH^high^/CD49f^high^ vs. ALDH^low^/CD49f^low^. (**F**) Quantification of tumor growth after 24 h *in vivo* on CAM. Dots represent the number of tumor cells per nodule. (**G**) Representative images of tumor nodules produced by ALDH^high^/CD49f^high^ and ALDH^low^/CD49f^low^ (left panel) or LRCs and NLRCs (right panel). T = tumor, H = host tissue (CAM), I = inoculation site. Scale bar: 8 mm. p-values estimated using one-way ANOVA followed by the Bonferroni correction with two-tailed *P* values<0.05 considered significant.

We next examined whether the ALDH^high^/CD49f^high^, LRC or CD49f^high^ sub-populations differed in their proliferative capacity *in vivo* from those that had lower levels of these markers. We sorted the T-HEp3 cells from nude mouse parental tumors into those with high or low CD49f levels (Fig. 4A, C and E) and/or ALDH activity (Fig. 4A, E and Fig. S3A). Proliferative capacity was monitored in the immunodeficient CAM model where HEp3 cells display high engrafting efficiency. This is a convenient way to monitor for proliferative capacity within 1 week [19], [20], [24]. Viable sorted HEp3 cells were inoculated at 10^2^–10^3^–10^4^ cells/CAM (20–2000 fold fewer cells than conventionally used [19], [41], [42]. We found that in all cases only CD49f^high^ (Fig. 4C), ALDH^high^ (Fig. S3A) or ALDH^high^/CD49f^high^ (Fig. 4 E–G) HEp3 cells displayed efficient proliferative potential *in vivo*, a difference detected with as little as 100 cells, meanwhile CD49f^low^ (Fig. 4C), ALDH^low^ (Fig. S3A) or ALDH^low^/CD49f^low^ (Fig. 4 E–G) HEp3 cells were inefficient in their ability to grow. Lack of growth of CD49f^low^, ALDH^low^/CD49f^low^ HEp3 cells was not due to dilution by host cells or due to effects of collagenase treatment on surface CD49f expression (Fig. S3B and data not shown). In addition, this difference was not attributable to differences in the adhesive capacity of CD49f^high^ and CD49f^low^ cells to fibronectin, collagen-I or laminin-I (Fig. S3C). This was paralleled by equal adhesion of CD49f^high^ and CD49f^low^ cells to the CAM tissue as 24 hrs after inoculation, there was no difference in the number of tumor cells recovered from the tissues (Fig. 4F). Thus, the enhanced proliferative capacity does not results from an enhanced adhesive potential due to CD49f enrichment that simply allows more cells to survive the *in vivo* inoculation.

**Figure 5:**
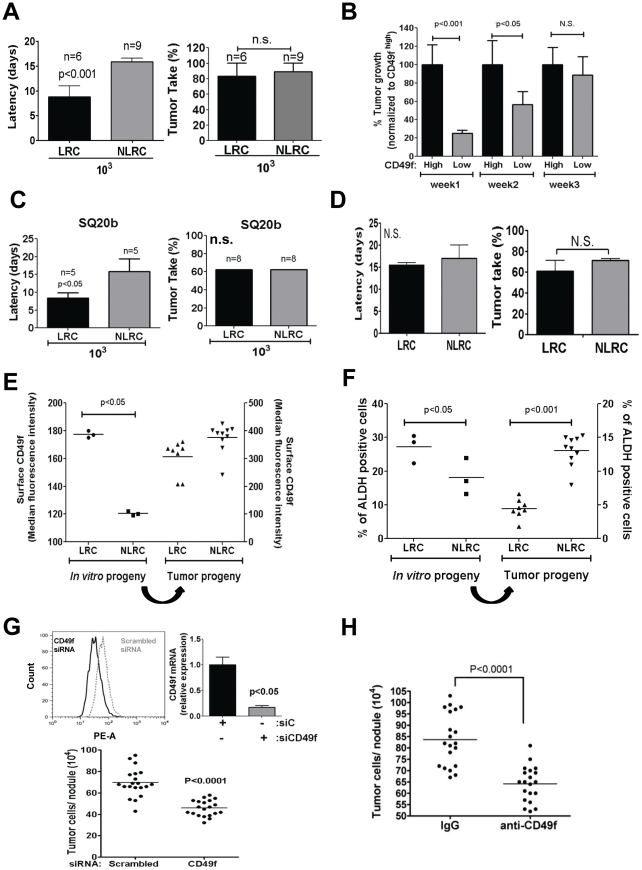
CD49f^low^/NLRCs can regain their tumorigenic capacity and restore CD49f and ALDH1A1 expression. (**A**) Tumor latency (left panel) and tumor take (Right panel) of HEp3 LRCs and NLRCs that were injected in nude mice (10^3^ cells/mice). Graphs show mean ± SD. The numbers represent the number of animals per group. (**B**) Quantification of tumor growth by CD49f^high^and CD49f^low^ HEp3 cells upon serial transplantation on CAMs (1–3 weeks). (**C**) Tumor latency (left panel) and tumor take (Right panel) of SQ20b LRCs and NLRCs that were injected in NSG mice with matrigel (10^3^ cells/mice). Graphs show mean ± SD. The numbers represent the number of animals per group. (**D**) Tumor latency (left) and tumor take (right) of the progeny of HEp3 LRCs and NLRCs after expansion in culture and injection in nude mice. (**E–F**) FACS analysis of surface CD49f (**E**) and ALDH1A1 (**F**) expression in the *in vitro* expanded progeny of LRCs and NLRCs (*in vitro* progeny) (Left part of the graph) and in tumors from the progeny of the LRCs and NLRCs expanded in culture (Tumor progeny)( Right part of the graph). (**G**) Quantification of HEp3 tumor growth after inhibition of CD49f using siRNA (Lower graph). Inhibition of CD49f expression was measured by FACs (upper left panel) and qPCR (upper right panel) (siC = SiRNA scrambled; siCD49f = siRNA CD49f). (**H**) Quantification of HEp3 tumor growth after treatment with a CD49f blocking antibody or an isotype matched IgG. p-values estimated using Mann-Whitney non-parametric test.

We also sorted HEp3 LRCs from NLRCs using CFSE label retention after one week in nude mice (Fig. 4B). These were inoculated at 10^3^ or 10^4^ cells/CAMs (Fig. 4D and G) or at 10^3^ cells/mouse in nude mice (Fig. 5A) with Matrigel. HEp3 LRCs inoculated in CAM were more efficient in proliferating *in vivo* (Fig. 4D) and similar to the tumor nodules produced by ALDH^high^/CD49f^high^, HEp3 LRCs also produced solid tumor nodules (Fig. 4G). In agreement with this, HEp3 LRCs injected in nude mice showed a faster tumor take and thus shorter latency phase than their NLRCs counterparts (Fig. 5A, left graph). When monitoring the populations of cells that did not grow (HEp3 marker-low or NLRCs) we found that regardless of the label used for sorting (ALDH, CD49f or CFSE) these surviving cells did not divide efficiently during the first week: non-tumorigenic (ALDH^low^/CD49f^low^ or NLRCs) 0.3±0.1 vs. tumorigenic (ALDH^high^/CD49f^high^ or LRCs) 0.86±0.1 population doublings/day (p<0.0001 t-test two tailed). We conclude that using three independent markers, we can identify an ALDH^high^/CD49f^high^ slow-cycling population in T-HEp3 tumors. These cells are endowed with proliferative capacity such that when sorted, they are capable of a rapid exit from their transient growth arrest, a strong and immediate proliferative response not observed in the marker^low^ (ALDH^low^/CD49f^low^) or NLRC subpopulation.

### CD49f^low^ cells or NLRCs Display a Latent Tumorigenic Potential

We next tested the *in vivo* long-term stability of the phenotype associated with ALDH^high^/CD49f^high^ or LRCs and ALDH^low^/CD49f^low^ or NLRCs subpopulations. If ALDH^high^/CD49f^high^ cells or LRCs are the only ones endowed with tumorigenic capacity these should be the only population showing transplantability *in vivo*. As shown above in the first week *in vivo* CD49f^high^/LRC cells sorted from nude mice tumors proliferated more efficiently than CD49f^low^/NLRC cells *in vivo* (Fig. 4C–D). However, upon serial transplantation in CAM we observed that by week 3, CD49f^low^ HEp3 cells had regained growth capacity (Fig. 5B). A similar behavior was observed for HEp3 NLRCs, by week 5 they had recovered their tumorigenic capacity (Fig. S4A). Likewise, when we injected 10^3^ HEp3 LRCs or NLRCs in nude mice, while LRCs had a shorter latency than NLRCs, we observed no difference in overall tumor take, indicating that tumorigenic potential is similar for HEp3 LRCs and NLRCs (Fig. 5A right and left panels). A similar behavior was observed for SQ20b LRCs and NLRCs sorted cells and injected in NSG mice (Fig. 5C). Thus, during the first 15–20 days, NLRCs, isolated from either HEp3 or SQ20b tumors and inoculated in mice or CAMs, show only a transient delay in exponential growth initiation but this does not impact their overall tumor take (Fig. 5A, B and C).

The restoration of tumorigenic potential was also observed after *in vitro* expansion of the HEp3 LRC and NLRC sorted subpopulations. All sorted subpopulations efficiently proliferated in culture (Fig. S4B and data not shown) and upon injection, all progenies from HEp3 LRCs and NLRCs were able to form tumors with similar efficiency in nude mice (Fig. 5D) or on CAM (Fig. S4C). Moreover, the ability of the progeny of NLRCs kept *in vitro* to regain tumorigenic capacity in those first two weeks *in vivo* was stable, as the tumors were able to grow again upon transplantation (Fig. S4C). The same behavior was observed for the progeny of the ALDH^high^ and ALDH^low^ HEp3 sorted populations that were expanded *in vitro* and then inoculated on CAM (Fig. S4D). To rule out the possibility that NLRCs were contaminated with LRCs that escaped detection we performed purity tests before and after sorting of the NLRC population (Fig. S5A). We also used double detection during the sort to avoid contaminating both fractions with cells that are non-specifically attached to each other. Using these procedures and stringent gates we found that the percent of positive cells in the negative fractions was as follows for three different labels (ALDH 0.5±0.05; CFSE 0.53±0.07; CD49f 0.5±0.25). However, because the sorted events may not always represent live cells at these low levels we visually inspected the sorted fractions *via* fluorescence microscopy. In all cases the negative fraction was fully devoid of marker-positive cells (data not shown). To further rule out that a contaminant of TICs might be responsible for regained tumorigenecity of the marker-negative cells, we calculated what would be the percent of the total tumor cell number contributed by these contaminating cells in weeks 1–3 for the CD49f (Fig. 5B) and weeks 1–7 for CFSE sorts (Fig. S4A). Based on these calculations we found that in the CD49f sort, CD49f^high^ cells contaminating the CD49f^low^ fraction would only account for 0.2, 4.0 and 4.2% of cells in those tumors in weeks 1, 2 and 3, respectively. In the CFSE sorts a similar result was obtained and after the 4^th^ week of propagation only 2.4–3.0% of the total tumor cells in the NLRC tumors would derive from contaminating CFSE+ cells. Thus, our results strongly support that the vast majority (95.8–99.5%) of the tumor growth produced by CD49f^low^ and NLRCs is due to the marker-low/negative cells. This further argues that although delayed in time marker-low/negative cells can be tumorigenic. In addition, in two independent sorts the progeny of HEp3 LRCs and NLRCs kept in culture retained CD49f^high^/ALDH^high^ and CD49f^low^/ALDH^low^ expression/activity (Fig. 5E–F and Fig. S5B upper panels), even after 100 passages *in vitro* (Fig. S5C) suggesting they are highly pure and stable (at least *in vitro*) marker^low^ populations. The same results were obtained when CD49f mRNA expression was measured by qPCR in HEp3 CD49f^high^ and CD49f^low^ progeny and in HEp3 LRC and NLRC progeny (Fig. S5D).

In stark contrast to the difference in marker levels observed *in vitro* (Fig. 5E–F) and that resembled the original sort, all tumors formed by HEp3 LRC and NLRC progeny displayed equal and high levels of surface CD49f as well as ALDH (Fig. 5E–F, Fig. S5B lower panels). In fact, HEp3 NLRC-derived tumors displayed even higher ALDH levels than LRCs (Fig. 5F). Thus, even HEp3 NLRCs that can stably maintain a CD49f^low^/ALDH^low^ profile in culture (Fig. 5E–F), are able to reactivate into a tumorigenic phenotype within 1–3 weeks *in vivo* (Fig. S4C) and this is associated with restoration of a CD49f^high^/ALDH^high^ profile (Fig. 5E–F) and regeneration of a LRC subpopulation (Fig. S5E). We conclude that although upon sorting, CD49f^low^/ALDH^low^ cells or NLRCs display diminished proliferative capacity they are not terminally committed to this phenotype. Instead these cells can regain tumorigenic capacity and this is associated with restoration of a CD49f^high^ and ALDH^high^ profile.

Furthermore, CD49f was required to support HEp3 tumor growth because downregulation of CD49f using a siRNA or blocking CD49f function with a function-blocking monoclonal antibody (clone GoH3) caused a significant reduction in HEp3 tumor growth (Fig. 5G–5H). It is unlikely that, the GoH3 antibody completely blocks adhesion of HEp3 cells to the ECM *in vivo* as HEp3 cells adhere poorly to laminin when compared to α5β1-integrin-mediated adhesion to fibronectin or adhesion to collagen-I [41], [43] (Fig. S3C). We conclude that CD49f activity in this system is functionally linked to HEp3 tumorigenicity.

### Phosphorylation of ERK^MAPK^ and Methylation of Histone-3 (H3) Tail in Intratumor LRCs and NLRCs

**Figure 6:**
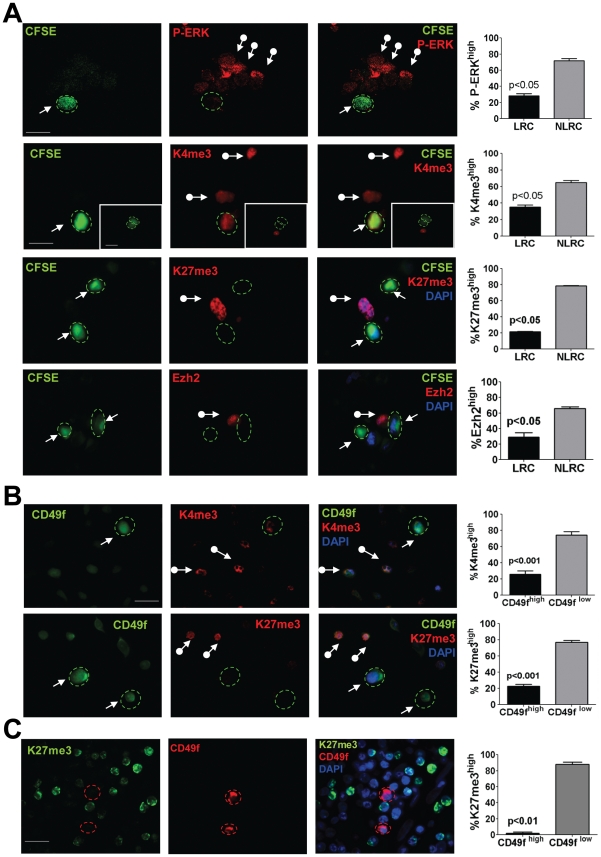
Characterization of ERK signaling and H3 post-translational modifications in TICs. (**A**) Detection of K27me3, K4me3, EZH2 and P-ERK1/2 by IF in CFSE-labeled HEp3 cells grown *in vivo* for 4 days. Representative images, LRCs stained for P-ERK (confocal scanning image – scale bar = 60 µm), K27me3, K4me3 and EZH2 (all standard fluorescence microscopy images – scale bar = 80 µm- scale bar inset = 60 µm). Contour of LRCs is delineated with a dashed green line. Arrows = LRCs, arrows with circle = NLRCs. Quantification of marker-positive or negative in LRCS and NLRCs is shown in the graphs on the right. Y axes show the percentage of cells per tumor. Graphs show mean ± SD of three independent tumors. (**B**) Detection of CD49f, K27me3 and K4me3 in HEp3 cells grown *in vivo* 6 days. Representative images of CD49f, K27me3 and K4me3, standard fluorescence microscopy images – scale bar = 80 µm- Contour of CD49f^high^ cells is delineated with a dashed green line. Arrows = CD49f^high^, arrows with circle = CD49f^low^. Quantification of marker-positive or negative in CD49f^high^ and CD49f^low^ cells is shown on the graphs on the right. Y axes show the percentage of cells per tumor. Graphs show mean ± SD of three independent tumors. (**C**) Detection of K27me3 and CD49f in sections from human oral primary tumors (PT68880). Scale bar = 80 µm. CD49f positive cells are delineated with a dashed red line. Y axes show the percentage of cells per section. p-values estimated using Mann-Whitney non-parametric test.

**Figure 7:**
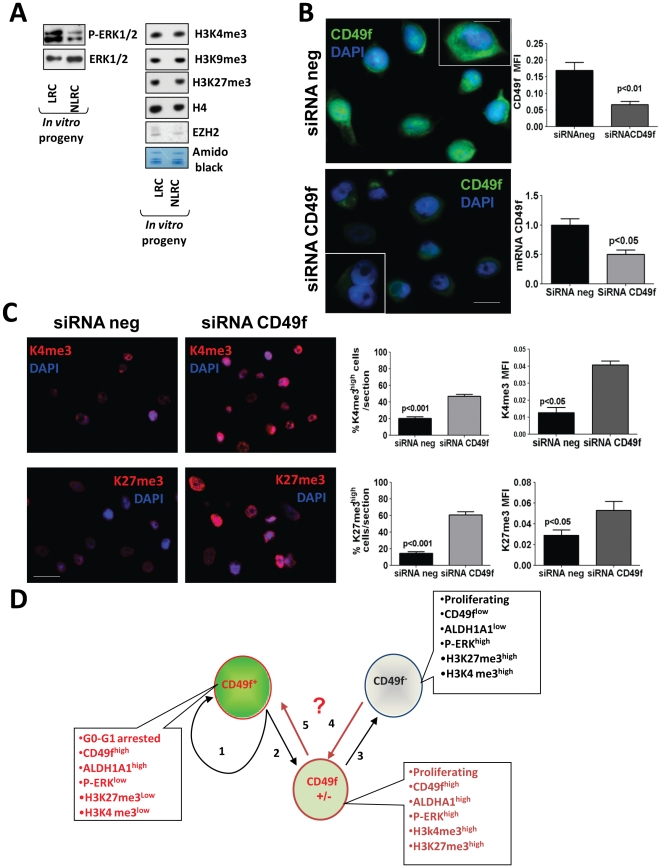
Characterization of H3 post-translational modifications upon knockdown of CD49f. (**A**) Immunoblot of phospho- and total-ERK1/2, H3K27me3, H3K4me3, H3K9me3 and EZH2 expression in LRCs and NLRCs sorted and expanded *in vitro*. Total ERK1/2 and Amido black were used as loading controls. (**B**) Inhibition of CD49f in HEp3 cells grown *in vivo* 6 days. Left panels: representative images of CD49f in HEp3 cells transfected with a scrambled siRNA (siRNA neg) or with a siRNA CD49f. The cells were permeabilized, thus CD49f signal bathes the whole cell but primarily the cytosol (scale bar = 60 µm). Insets, overlay of CD49f and DAPI, showing the CD49f characteristic membrane and cytosolic staining (Scale bar = 40 µM). Upper right graph- quantification of CD49f mean fluorescence intensity (MFI). Lower right graph,-quantification of CD49f expression by qPCR. (**C**). Representative images of K4me3 and K27me3 in HEp3 cells transfected with a scrambled siRNA (siRNA neg) or with a siRNA CD49f. Left panels- standard fluorescence microscopy images – scale bar = 80 µm. Quantification of K27me3^high^ and K4me3^high^ percentage of cells and mean fluorescence intensity is shown on the graphs on the right. Graphs show mean ± SD of three independent tumors. p-values estimated using Mann-Whitney non-parametric test. (**D**) Scheme depicting the hypothetical behavior of different populations in HNSCC tumors. Marker high (CD49f^+^) and marker low (CD49f^−^) populations within HNSCC tumors can be defined by their corresponding programs characterized by the markers indicated in the dialogue boxes. CD49f+ can self-renew (path 1) or after transiting through an intermediary state (CD49f^+/−^) (Path 2) fully reprogram into CD49f^−^ (Path2+3). The intermediary state (CD49f^+/−^) is defined by default by the markers in the dialogue box. The MTP model proposed that CD49f^−^ cells can also transit through the intermediary state (Path 4) to then fully reprogram into CD49f^+^ (Paths 4+5). Our model also considers that the different paths are stochastic and tumor cells might transition back and forth between the different states. A remaining open question is what controls the dynamic plasticity that drives the alternation between these states (question mark).

Our data revealed a dynamic heterogeneity in the cycling capacity of marker defined subpopulations in HNSCC experimental tumors, a characteristic that seems associated with the timing of tumorigenic capacity. These different behaviors might have a functional role during tumor growth. Thus, we characterized how HNSCC cells transit into and out of the slow-cycling CD49f^high^ phenotype. Using an Elk-GAL4::GFP reporter we previously showed that ERK1/2 activity is high in growing HEp3 tumors, but not all HEp3 cells activated ERK [19], suggesting that not all cells within the tumor were actively exiting G0–G1. Thus, we explored the levels of ERK1/2 phosphorylation directly in HEp3 LRCs *in situ*. Staining for CFSE and P-ERK1/2, revealed that while ∼70% of NLRCs showed strong P-ERK1/2 signal only 20% of the LRCs showed ERK1/2 activation (Fig. 6A upper panel). Thus, the slow-cycling behavior of LRCs correlated with reduced ERK1/2 activation. We next tested P-ERK1/2 levels in HEp3 sorted LRCs, because immediately after sorting these P-ERK1/2^low^ cells proliferate efficiently (Fig. 4D). Western blot for P-ERK1/2 and total ERK1/2 levels revealed that the progeny of LRCs actively proliferating in culture displayed stronger ERK1/2 phosphorylation than the progeny of NLRCs; total ERK1/2 levels remained unchanged (Fig. 7A). In culture the proliferation of LRCs and NLRCs was indistinguishable (data not shown), suggesting that the main change is a burst in ERK1/2 activation upon sorting of LRCs that were slow cycling or arrested in the tumor. We conclude that in LRCs, the transient growth arrest and low ERK1/2 phosphorylation are dynamically regulated and that sorting relieves a break on ERK1/2 activity that correlates with active proliferation.

The data above suggest that HEp3 LRCs are poised for immediate expansion upon sorting (Fig. 4D) or within tumors in response to an unidentified signal that activates their G0–G1 exit *in vivo*. HEp3 NLRCs are also poised for growth (Fig. 5A–C), but apparently must undergo a “reprogramming” phase that takes longer (15–21 days) (Fig. 5 A–C). A similar scenario was reported for embryonic stem cells that have their chromatin in a “bivalent” state that must be resolved upon receiving specific stimuli to differentiate [44]. Recently, high levels of a histone 3 (H3) lysine-4 (H3K4) demethylase JARID1B were shown to define a slow-cycling sub-population important for melanoma tumorigenesis [45]. Despite the use of two different antibodies, we could not find differences in JARID1B staining between HEp3 LRCs and NLRCs *in situ* (Fig. S6A). However, we did find that only ∼35% of HEp3 LRCs were H3K4me3^high^ (a target of JARID1B) while ∼65% of NLRCs were high for this mark that is associated with transcriptional activation (Fig. 6A). We also tested the levels of H3K4me3 in HEp3 CD49f^high^ and CD49f^low^ cells (Fig. 6B) and found that similar to NLRCs, 75% of HEp3 CD49f^low^ cells were H3K4me3^high^. This suggests that the LRC/CD49f^high^ population may have a unique chromatin state and perhaps other H3 PTMs might play a role in determining LRC/CD49f^high^ to NLRC/CD49f^low^ switch and *vice versa*. We next measured the levels of H3K27me3 and H3K9me3 marks that are associated with facultative and constitutive heterochromatin, respectively [46]. These marks have also been found to be modulated in teratocarcinomas and adult bulk cultures of colon cancer cells [44]. We found that close to 80% of HEp3 NLRCs/CD49f^low^ displayed strong nuclear staining for H3K27me3, while not more than 20% of HEp3 CD49f^high^/LRCs were H3K27me3^high^ (Fig. 6A–B). In addition, 80% of NLRCs were H3K9me3^high^ (Fig. S6B). DAPI staining was used to conclusively identify the larger nuclei in HEp3 cells as described [19]. The 20% of CD49f^high^/LRCs HEp3 cells were not entirely negative for these H3 PTMs, but rather the signals were low and less abundant (Fig. S6C). Analysis of total H3 expression by immunofluorescence showed that all cells in both populations were positive for total H3 with equal intensities (Fig. S6D).

In agreement with the reduced H3K27me3 staining, *in situ* detection of EZH2, the catalytic subunit of the PRC2 complex that methylates H3K27 [47], showed that this enzyme was also low (not absent) in HEp3 LRCs (Fig. 6A). Further, after sorting, the growing HEp3 LRC progeny restored the levels of EZH2 expression, which is consistent with this enzyme being an E2F target gene during G1 exit [48] (Fig. 7A).

Because CD49f is required for HEp3 tumor growth (Fig. 5G–H), we tested the possibility that H3 PTMs might be downstream of CD49f signaling. Thus, we examined whether the levels of H3K27me3 and H3K4me3 were modulated upon inhibition of CD49f (Fig. 7B). We found that inhibition of CD49f caused a strong overall increase of H3K4me3 and H3K27me3 marks (Fig. 7C). These results suggest that there is a functional link between CD49f and H3K4me3 and H3K27me3 marks. However, we didn't see any effect on EZH2 expression when we knockdown CD49f, at as least measured by IF.

Importantly, in agreement with the findings in HEp3 CD49f^high^/LRCs, we found that in patient primary HNSCC tumors CD49f^high^ cells displayed almost exclusively a H3K27me3^low^ profile (Fig. 6C). We conclude that the H3K27me3 repressive mark is consistently less abundant in CD49f^high^/LRC cells in both HEp3 and patient HNSCC tumors. This suggests that CD49f^high^/LRC might have a chromatin state poised for active transcription. The progeny of sorted CD49f^high^/LRC expanded in culture showed that although ERK1/2 activation and H3K27me3 and H3K9me3 were upregulated, they also retained high CD49f expression (Fig. 7A, Fig. 5E and Fig. S5B, C, D). The correlative nature of these studies only allows us to conclude that the behavior of CD49f^high^/P-ERK^low^/H3K4/K27me3^low^ cells may be governed by epigenetic mechanisms, allowing them to exist in a dynamic state that dictates tumorigenic capacity.

## Discussion

The identification of tumor cell subpopulations in HNSCC tumors with dynamic tumorigenic potential raises several important questions. Our studies suggest that both CD49f^high^ cells and LRCs and CD49f^low^ and NLRC cells are highly dynamic in their phenotypes allowing the tumor as a whole to remain adaptable to growth needs and perturbations of the different subpopulations. Our findings do not completely fit a strict hierarchical model definition for tumor initiating cells. In fact, our data may better fit a model where epigenetic and micro-environmental factors dynamically regulate the phenotypic heterogeneity of different subpopulations that perhaps dynamically establish a hierarchy where CD49f^high^/LRC cells are at the top of the hierarchy and CF49f^low^/NLRC are the “differentiated” progeny. The dynamic and reversible occurrence of these defined subpopulations (i.e. CD49f^high^/LRCs) suggests that through this phenotypic heterogeneity some tumors may activate a transient and reversible structure to propel tumor expansion. This has been proposed in other systems [18], [49] and it is evidenced by the capacity of NLRCs and CD49f^low^ cells to regain CD49f^high^/ALDH^high^ expression, regenerate the slow-cycling population and recover tumorigenic capacity.

Here we provide a molecular description of these subpopulations and reveal a previously unrecognized association between this dynamic phenotypic heterogeneity and changes in epigenetic markers. We determined that even the marker-low subpopulation (NLRCs/CD49f^low^) survives the engraftment (10^2^–10^3^ cells) and is able to regenerate a marker-high subpopulation (i.e. CD49f^high^) with tumorigenic potential. This is in agreement with other studies suggesting non-hierarchical and dynamic mechanisms driving iPS cell reprogramming [50] and the behavior of melanoma tumor cell sub-populations with reversible tumorigenic phenotypes [45]. Our results also resemble recent findings showing that transcription factors that cause epithelial-to-mesenchymal transition (EMT) can reversibly induce stem cell-like properties in bulk populations of mammary tumor cells [51].

We believe that the identification of tumor cell subpopulations with a dynamic regulation of their phenotypic heterogeneity might have important implications for therapy at least in HNSCC. Even if marker-defined cells (CD49f^high^/ALDH^high^) fit the TICs definition and these tumor cells were to be specifically targeted and eradicated, the residual microscopic disease (CD49f^low^/ALDH^low^) not eliminated by debulking therapies could give origin to a secondary lesion. Thus, if those cells that escape debulking strategies may regain a TIC phenotype then eliminating TICs to irreversibly curtail tumor growth might need of more persistent strategies to block “TIC” properties [52].

The detection of a subpopulation of CD49f^high^/H3K27me3^low^ cells in human primary tumors, although descriptive, strengthens our findings and their relevance to human disease. In human tumors sections, CD49f^high^ cells were found as single solitary cells or as small clusters displaying very strong CD49f signal and both the frequency and overall staining intensity increased in metastatic lesions. CD49f is a laminin-5 binding integrin (α6-integrin) that pairs with β4 and β1 integrin subunits to regulate adhesion signaling [53], [54], but can also signal in a laminin independent manner [55]. While CD49f was clearly contributing to the tumorigenic capacity of HEp3 tumor cells and also maintaining low H3K27me3 levels, how CD49f function is linked to the transient arrest and subsequent recruitment of these cells for expansion is not clear. In the bulk of HEp3 tumors α5β1 integrins are activated by the urokinase receptor (uPAR) to recruit focal adhesion kinase and EGFR to activate ERK1/2 signaling and tumor growth [42]. Recent studies suggest that uPAR might function as a self-renewal regulator and that by pairing with α6-integrin [56]. Also uPAR expression has been shown by others and us to follow an oscillating and dynamic pattern of expression [57]. Thus, although it remains unknown, it is possible that reversible regulation of uPAR and CD49f expression might contribute to the LRC and NLRC behavior.

The ability of CD49f^low^ cells and NLRCs to regenerate a CD49f^high^ and LRC subpopulations, respectively, suggested that epigenetic programs might drive the shift between these states. A similar scenario is observed in embryonic stem (ES) cells that appear to be poised for active transcription upon receiving specific stimuli [58], 59,60. Characterization of the CD49f^high^ and LRCs revealed that consistent with their transient growth-arrest, they display low ERK1/2 phosphorylation. However, upon sorting these cells are immediately able to rapidly proliferate. This correlated with a burst in ERK1/2 activation and suggests that these cells are primed to respond to currently unidentified activating signals for expansion. Analysis of H3 post-translational modifications showed that 65–80% of CD49f^high^/LRCs were H3K4me3^low^, H3K9me3^low^ and H3K27me3^low^ (not negative), with the latter two repressive marks [58], [59], being more dramatic in their decrease. This correlated with a similar distribution for EZH2^low^ levels in LRCs. These data suggest that CD49f^high^ cells and LRCs, which are also CD49f^high^, may be held in a transcription-ready state due to their low levels of transcriptionally repressive marks. The NLRC and CD49f^low^ subpopulation may have a chromatin organization that reflects a bivalent state, having to remove K27me3 in order activate the strong proliferative program observed in LRCs.

Finally, we found similar proportion of JARID1B^high^ cells in LRCs and NLRCs. This contrasts recent reports [45] where JARID1B enriched cells define a slow-cycling melanoma subpopulation. While the use of different methods to identify the slow–cycling cells might explain this difference, based on our quantification, there is a small fraction of CFSE+ cells that are JARID1B^high^/H3K4me3^low^ and H3K27me3^high^/H3K9me3^high^. This subpopulation of cells may have a highly repressive chromatin state, but their fate and function is currently unclear.

As mentioned above, majority of LRCs (∼80%) were globally H3K4me3^low^ and H3K9/K27me3^low^. In contrast, NLRCs displayed primarily global repressive (H3K4me3^low^ and H3K27me3^high^/H3K9me3^high^) or bivalent H3K4me3^high^ and H3K27me3^high^/H3K9me3^high^) chromatin states. A reversion to a more permissive state for transcription that might require erasing and/or writing new H3 PTMs might explain why longer periods are needed for NLRCs to fully restore their full tumorigenic potential. *In vitro* expanded LRCs reactivated ERK1/2 and upregulated EZH2. However, they retained a “memory” of the high CD49f levels in culture relative to NLRCs. This suggests that in LRCs, activation of ERK1/2 signaling and EZH2 expression is not key in regulating CD49f expression. Whether the above H3 marks are present/absent in the promoter region of CD49f gene in order to regulate its expression is unknown. However, the opposite seems to be true, as RNAi to CD49f cause a global increase in H3K27me3^high^ cells, suggesting that regulation of H3 PTMs are downstream from signaling by this integrin. While mechanistically unclear, such signaling to chromatin is intriguing and warrants future study. It also remains unclear what the functional relevance of the slow-cycling behavior of LRCs shown here and by others is [45]. Analysis of these subpopulations directly isolated from fresh human tumors and implanted in NSG mice may provide additional insight into the relevance of our findings.

Our work identifies a novel mechanism controlling a dynamic tumorigenicity program in HNSCC experimental tumors models. Recently it was shown that chromatin remodeling can lead to adaptive (rather than selective) mechanisms that activate a drug-tolerant phenotype in cancer cells [61]. Thus, a better understanding of the mechanisms regulating the phenotypic heterogeneity of tumor cell subpopulations might lead to strategies that efficiently block the adaptive capacity of tumors to microenvironment- and/or therapy-derived stress conditions.

## Materials and Methods

### Ethics Statement

All animal studies were approved by Institutional Animal Care and Use Committees (IACUC) at Mount Sinai School of Medicine (MSSM). Protocol ID: 08-0728. Start: 06-Apr-2009. Title IACUC protocol: Plasticity of head and neck cancer initiating cells.

### Cell Lines and Xenograft Studies

Tumorigenic (T-HEp3) HEp3 cell were described previously [26]. Tumor growth on chick embryo CAMs or Balb/c nude mice has been described previously [41]. All animal experiments were approved by IACUC (MSSM). When studying the effect of the function-blocking CD49f antibody in vivo, 1×10^6^ T-HEp3 cells were resuspended in 250 µl PBS^++^ and incubated with either Isotype Control Rat IgG or CD49f blocking antibody (10 µg/ml) for 30 minutes at 37°C. 2×10^5^ cells were inoculated onto each CAM. Inoculated cells were treated 24 h post inoculation on the CAM with the antibodies. Tumors were harvested 4 days later, minced, digested with collagenase-1A and the number of tumor cells per nodule was counted. For serial transplantation studies of HEp3 tumors on CAM, HEp3 tumors were minced and dissociated with collagenase-IA (see Flow cytometry for additional details of tumor processing). Tumor cells were counted and a single cell suspension (8×10^4^ cells in 50 µl PBS) from each tumor was reinoculated on CAMs. For RNA interference studies, transfections of cells with siRNAs targeting the desired sequences or control scrambled siRNA were performed as previously described [14]. 24 h after transfection 2×10^5^ cells were inoculated on the CAM of 9–10-day-old chicken embryos (Charles River). One-week post inoculation, the number of tumor cells per nodule was counted. siRNA knockdown was analyzed either by qPCR or FACS.

### Flow Cytometry

Single-cell suspensions were generated from HEp3, SQ20b or FaDu xenografts by surgical dissection of tumors from euthanized nude mice or chicken embryo chorioallantoic membranes (CAMs) (when indicated for the HEp3s). Tumors were excised, weighed, minced and enzymatically dissociated into single cell suspensions by incubation with type 1A collagenase (Sigma - C9891) for 30 min at 37°C. The suspension was then homogenized by pipeting and the collagenase was inactivated with full serum media. Subsequently, tumor cells were washed with media for excess collagenase removal. Tumor cells, recognized by their very large diameter, were counted with a hemocytometer. Cell viability was determined by trypan-blue exclusion. Then tumor cells were enriched using a Percoll gradient [41], [62] to remove host cells. ALDH1A1 activity was detected using Aldefluor reagent (Stem Cell Technologies Inc) according to manufacturer's instructions. In some experiments, cells were co-stained with either phycoerythrin-conjugageted CD49f or CD44 antibodies. A list of primary antibodies is in Methods S1. Samples were analyzed using an LSR II instrument (Becton Dickinson). Size-based gates were used to gate out host cells (Chicken or mouse cells), that are smaller than HEp3 cells. A PE-conjugated Isotype-matched mouse antibody (R&D Systems) was used as a control. For cell cycle analysis studies, T-HEp3 cells were stained for ALDH and CD49f as described above and then cells were stained using Hoechst-33342 (10 µg/ml) for 15 min at 4 C. The cells were washed, centrifuged and analyzed. For cell sorting experiments, T-HEp3 or SQ20b cells were harvested either from nude mice or CAMs (in the case of HEp3), processed as described above and sorted on a FACSAria Cell Sorter (Becton Dickinson, San Jose, CA). Sorting was performed at low pressure and using a 120 or 150 µm nozzle to maximize the viability of sorted cells. In all sorts all doublets and larger cell aggregates were excluded and only single cells were sorted. After sorting, an aliquot of sorted cells was always reanalyzed to check for purity, which was usually >95. Tumor cells were then counted and resuspended in PBS^++^ Viable cells were inoculated at 10^2^, 10^3^, or 10^4^ cells/50 µl either on CAM (HEp3 cells) or subcutaneously in mice (nude mice for HEp3 cells and NGS mice for SQ20b cells). Tumors growth on CAM was assessed at 7 days. Tumor formation in nude or NGS mice was evaluated regularly after injection by palpation of injection sites, and tumor diameters were measured with a caliper.

### Label Retention Assay

T-HEp3 cells were incubated with CFSE (20 µM) in PBS for 15 min at 37°C degrees and then were allowed to recover in complete media for 30 min prior to injection in nude mice or CAMs. Cells were allowed to grow *in vivo* for different periods of time (4–6 days in CAM, 8–12 days in nude mice). Cells from these tumors were processed as described in the flow cytometry section above and CFSE+ population (LRC) was analyzed by flow cytometry and fluorescence microscopy.

### Immunohistochemistry

Paraffin embedded sections from human primary tumors and lymph node metastasis were stained for CD49f expression after quenching endogenous peroxidase activity. Binding of the primary antibody was carried out for 30 min at room temperature, detected by anti-rat secondary (30 min at room temperature) and revealed using DAB Chromagen.

### Immunofluorescence

Cytospins and tumor cells seeded in coverslips were fixed with 4% paraformaldehyde (PFA) in PBS and were permeabilized with 0.1% Triton X-100 (+0.01% SDS in the case of the K27me3, K4me3, EZH2 and K9me3 staining). Cells were then blocked with 1% PBSA containing 3% normal goat serum (NGS). CD49f staining did not require a permeabilization step. A list of primary antibodies is in Methods S1. Secondary antibodies used for immunofluorescence microscopy were: goat anti-rabbit Alexa Fluor 568, goat anti-rabbit Alexa Fluor 488 and goat anti-mouse Alexa Fluor 488 (Invitrogen). For fluorescence analysis of CFSE stained samples, tumor cells were fixed in 4% PFA and either seeded in coverslips pretreated with polylysine or used to prepare cytospins. Cytospins were prepared according to standard protocols.

### Immunoblotting, RT-PCR and Quantitative qPCR

Immunoblotting and RT-PCR were performed as described previously [14]. Two micrograms of total RNA isolated from HEp3 cells (Trizol reagent, Invitrogen) were reverse-transcribed using MMuLV RT (NEB, Ipswich, MA) and then amplified by standard PCR using Taq DNA polymerase (NEB, Ipswich, MA) following manufacturer's instructions. Primers were purchased from IDT (Coralville, IA). Primer sequences for CD49f are, 5′ ATGGAGGAAACCCTGTGGCT 3′ (forward) and 5′ ACGAGAGCTTGGCTCTTGGA 3′ (reverse).

### Statistical Analysis

For the FACs and immunofluorescence assays the p-values were estimated using Mann-Whitney non-parametric test with two-tailed *P* values<0.05 considered significant. For primary and secondary tumor formation statistically significant differences were assessed using one-way ANOVA followed by the Bonferroni correction with two-tailed *P* values<0.05 considered significant.

## Supporting Information

Figure S1 **Expression of ALDH1A1, CD49f, CD44, and CD71 in HEp3 and SQ20b tumors.** (**A**) FACS quantification of CD49f expression in 2 HNSSC cell lines kept in culture (SQ20b and FADU) and *in vivo* maintained HEp3 cells. (**B–C**) FACS analysis of CD44 (B) or CD71 (C) expression in the ALDH^high^ and ALDH^low^ subpopulations. Right - representative histograms, with numbers indicating the CD44 (B) or CD71 (C) mean fluorescence intensity (MFI). Left - quantification of CD44 (B) or CD71 (C) MFI in at least 3 different HEp3 tumors. (**D**) Image of a cytospin from a CFSE labeled T-HEp3 tumor grown on the CAM for 4 days. The LRCs (green) are marked by arrows. Scale bar 40 µm. (**E**) Detection of LRCs (green) and CD49f positive cells (red) by immunofluorescence in CFSE-labeled HEp3 cells grown *in vivo* for 4 days. Scale bar 40 µm. Lower right corner inset: CFSE staining, the points mark the LRCs. Scale bar: 80 µm. Upper left corner inset: CD49f staining, the stars mark the CD49f^high^ cells. Scale bar: 80 µm. (**F**) Quantification of LRCs in SQ20b tumors grown in mice between 7 and 15 days. Left panel. each point represents the percentage of LRCs per tumor. Middle panel- quantification of LRCs in SQ20b tumors with different volumes grown in nude mice. The bigger the tumor is, the more the cells have divided, the more the CFSE is diluted. Therefore, the bigger tumors have lower percentage of LRCs. Right panel- Fluorescence photomicrograph of a SQ20b LRC (arrow) in a ∼1000 mm3 tumor. Scale bar: 80 µm.
(TIF)

Figure S2 **Expression of CD49f in normal epithelium. Representative images of CD49f staining in benign adjacent oral squamous epithelium of patient 68880 (A) and HEp3 tumor (B).** Scale bar: 20 µm (left panel) and 40 µm (right panel) in (**A**). Scale bar: 60 µm (**B**). (**C**) Table showing the quantification of the mean percent of CD49f positive cells in the primary tumor and metastasis from each patient. Right panel, graph representing the mean percentage of CD49f positive cells in human oral primary tumors (PT) and lymph node metastasis (met). p-values estimated using Mann-Whitney non-parametric test.
(TIF)

Figure S3 **CD49f^High^ and CD49f^Low^ cells have similar adhesive potential to the ECM.** (**A**) Quantification of tumor growth after 1 week *in vivo* of sorted ALDH^high^ and ALDH^low^ populations. Dots represent the number of tumor cells per nodule. p-values estimated using one-way ANOVA followed by the Bonferroni correction with two-tailed *P* values<0.05 considered significant (**B**) FACS quantification of CD49f mean fluorescence intensity in HEp3 tumors disaggregated with either collagenase or PBS/EDTA. (**C**) Adhesion to Fibronectin (FN), collagen (CL) and Laminin (LN) in HEp3, CD49f^high^ and CD49f^low^ cells. 25*10∧4 cells per well of 96-well plates, (four wells per experimental point) were inoculated into wells coated with FN 4 mg/ml, CL 4 mg/ml or LN 5 mg/ml incubated at 37 C for 30 min, fixed, and stained with crystal violet (see Methods S1), the dye was extracted, and the absorbance was measured at 570 nm.
(TIF)

Figure S4 **CD49f^low^/NLRCs can reprogram and regain their tumorigenic capacity.** (**A**) Quantification of the number of population doublings *in vivo* of LRCs and NLRCs upon serial transplantation for 5–7 weeks. (**B**) Detection of CFSE in HEp3 cells before sort (left column), immediately after sort (middle column) and 5 days after expansion *in vitro* (right column). Scale bar 120 µm. (**C**) Upper table, tumor take for the *in vitro* expanded progeny of LRCs and NLRCs on CAM after 1 week *in vivo*. Lower panel graph - LRC and NLRC produced tumors after 3 weeks are able to regenerate again tumors with equal efficiency after 1 week. (**D**) Quantification of tumor volume produced by the progeny of ALDH^high^ (ALDH+) and ALDH^low^ (ALDH−) and mixed populations (ALDH+/−) after *in vitro* expansion and reinoculation on CAM. Note that all cells produce tumors of similar size after being expanded *in vitro* for several weeks. p-values estimated using Mann-Whitney non-parametric test.
(TIF)

Figure S5 **Stability of CD49f expression and ALDH activity in the progeny of HEp3 LRCs and NLRCs in culture.** (**A**) Representative Histograms of purity controls after sort for NLRCs (right panel) or CD49f^low^ cells (left panel). (**B**) FACS quantification of CD49f (upper and lower left panels) and ALDH1A1 activity (upper and lower right panels) expression in the progeny of LRCS and NLRCs expanded *in vitro* after sorting (upper panels) from an independent set of tumors from Fig. 5 and after *in vitro* expanded cells were allowed to form tumors *in vivo* (lower panels). (**C**) FACS quantification of CD49f (left panel) and ALDH1A1 (right panel) expression in cells that after sorting were expanded *in vitro* for more than 100 generations. (**D**) Quantification of CD49f expression by qPCR in cells that after CD49f sorting were expanded *in vitro* for 6 passages (left panel) or that after CFSE sorter were expanded *in vitro* for 100 passages (right panel). (**E**) The tumor progeny of the NLRCs expanded in culture were labeled with CFSE (20 µM) and inoculated *in vivo*. NLRC after expansion *in vitro* regain tumorigenic capacity and are able to generate a LRC population that can be detected by fluorescence microscopy (left panel). Scale bar: 80 µm. Right panel, quantification of the number of CFSE positive cells in 4 days tumors formed by the progeny of the NLRCs.
(TIF)

Figure S6 **Jarid1B and H3K9me3 expression in HEp3 LRCs and NLRCs.** (**A–B**) Detection of CFSE, JARID1B (**A**) and H3K9me3 (**B**) by immunofluorescence in CFSE-labeled HEp3 cells grown *in vivo* for 4 days. Scale bar: A = 40 µm, B = 60 µm. Quantification of positive cells is shown on the right. Graph shows mean ± SD of three independent tumors. Y axes represent the percentage of cells per tumor. p-values estimated using Mann-Whitney non-parametric test. (**C**) Detection of CFSE, H3K27me3, H3K9me3, H3K4me3 by immunofluorescence. Here we used a short integration time on the digital camera to reveal the H3PTM high marks (second column). In the third column we used a longer integration time on the digital camera to show that H3-PTM marks are present in those cells that appear negative in the second column. This reveals that it is a degree difference and not a positive vs. negative difference. Scale bar: 80 µm. (**D**), Detection of total H3 levels in LRCs and NLRCs. Note that all cells are positive for total H3. Scale Bar = 80 µm.
(TIF)

Methods S1 **Adhesion assays.** Adhesion to Fibronectin (FN), collagen (CL) and Laminin (LN). HEp3, CD49f^high^ and CD49f^low^ cells were inoculated into wells coated with FN 4 mg/ml, CL 4 mg/ml or LN 5 mg/ml, incubated at 37 C for 30 min, fixed, and stained with crystal violet, the dye was extracted, and the absorbance was measured at 570 nm. Table of Antibodies.
(DOCX)
