# Supplementary figures and images for: Analysis of Marker-Defined HNSCC Subpopulations Reveals a Dynamic Regulation of Tumor Initiating Properties
Source: PLoS One. 2012 Jan 20;7(1):e29974. doi: 10.1371/journal.pone.0029974 (PMC3262798; doi:10.1371/journal.pone.0029974)

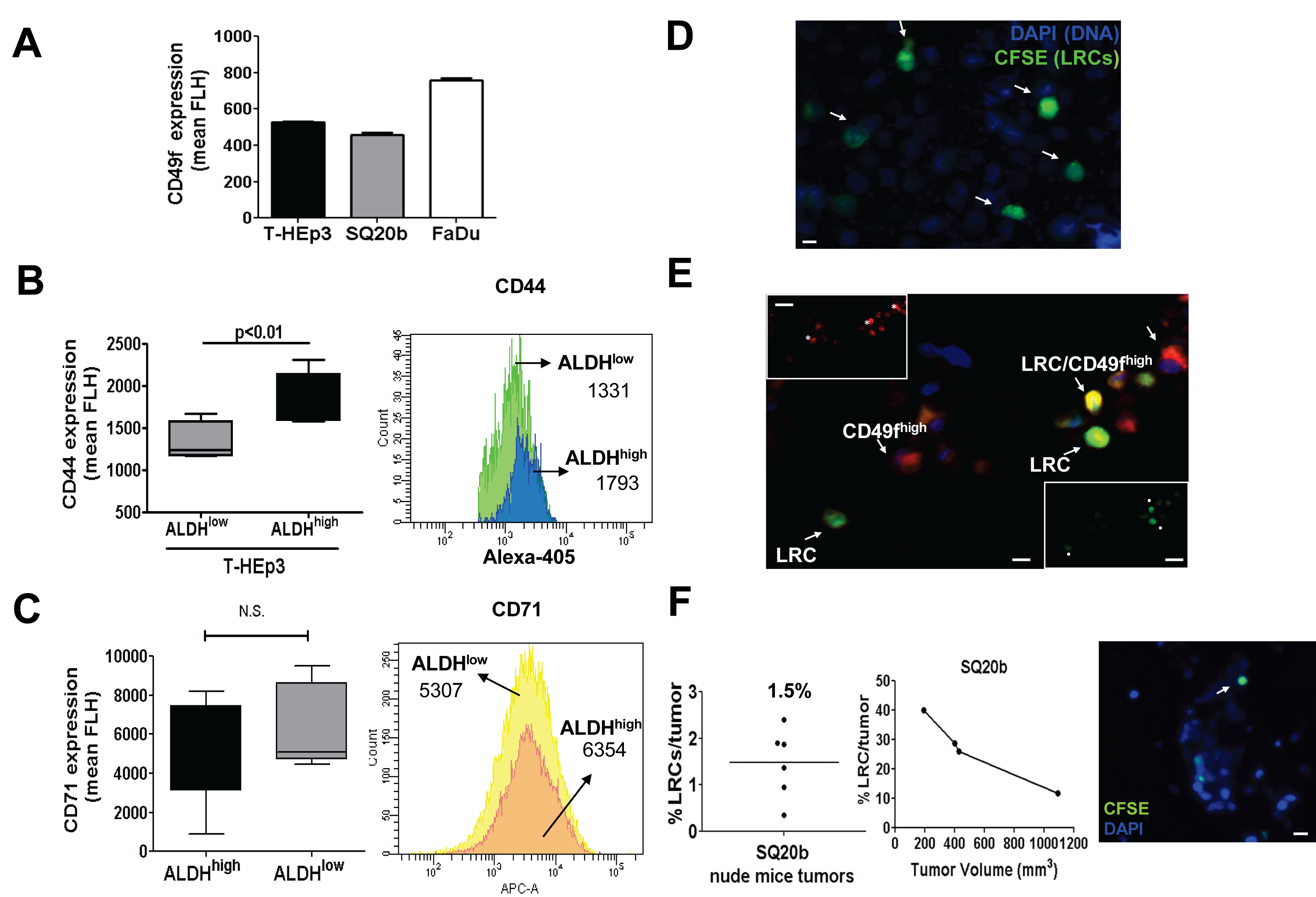

Supplement: Figure S1 — Expression of ALDH1A1, CD49f, CD44, and CD71 in HEp3 and SQ20b tumors. (A) FACS quantification of CD49f expression in 2 HNSSC cell lines kept in culture (SQ20b and FADU) and in vivo maintained HEp3 cells. (B–C) FACS analysis of CD44 (B) or CD71 (C) expression in the ALDHhigh and ALDHlow subpopulations. Right - representative histograms, with numbers indicating the CD44 (B) or CD71 (C) mean fluorescence intensity (MFI). Left - quantification of CD44 (B) or CD71 (C) MFI in at least 3 different HEp3 tumors. (D) Image of a cytospin from a CFSE labeled T-HEp3 tumor grown on the CAM for 4 days. The LRCs (green) are marked by arrows. Scale bar 40 µm. (E) Detection of LRCs (green) and CD49f positive cells (red) by immunofluorescence in CFSE-labeled HEp3 cells grown in vivo for 4 days. Scale bar 40 µm. Lower right corner inset: CFSE staining, the points mark the LRCs. Scale bar: 80 µm. Upper left corner inset: CD49f staining, the stars mark the CD49fhigh cells. Scale bar: 80 µm. (F) Quantification of LRCs in SQ20b tumors grown in mice between 7 and 15 days. Left panel. each point represents the percentage of LRCs per tumor. Middle panel- quantification of LRCs in SQ20b tumors with different volumes grown in nude mice. The bigger the tumor is, the more the cells have divided, the more the CFSE is diluted. Therefore, the bigger tumors have lower percentage of LRCs. Right panel- Fluorescence photomicrograph of a SQ20b LRC (arrow) in a ∼1000 mm3 tumor. Scale bar: 80 µm. (TIF) [file pone.0029974.s001.tif]

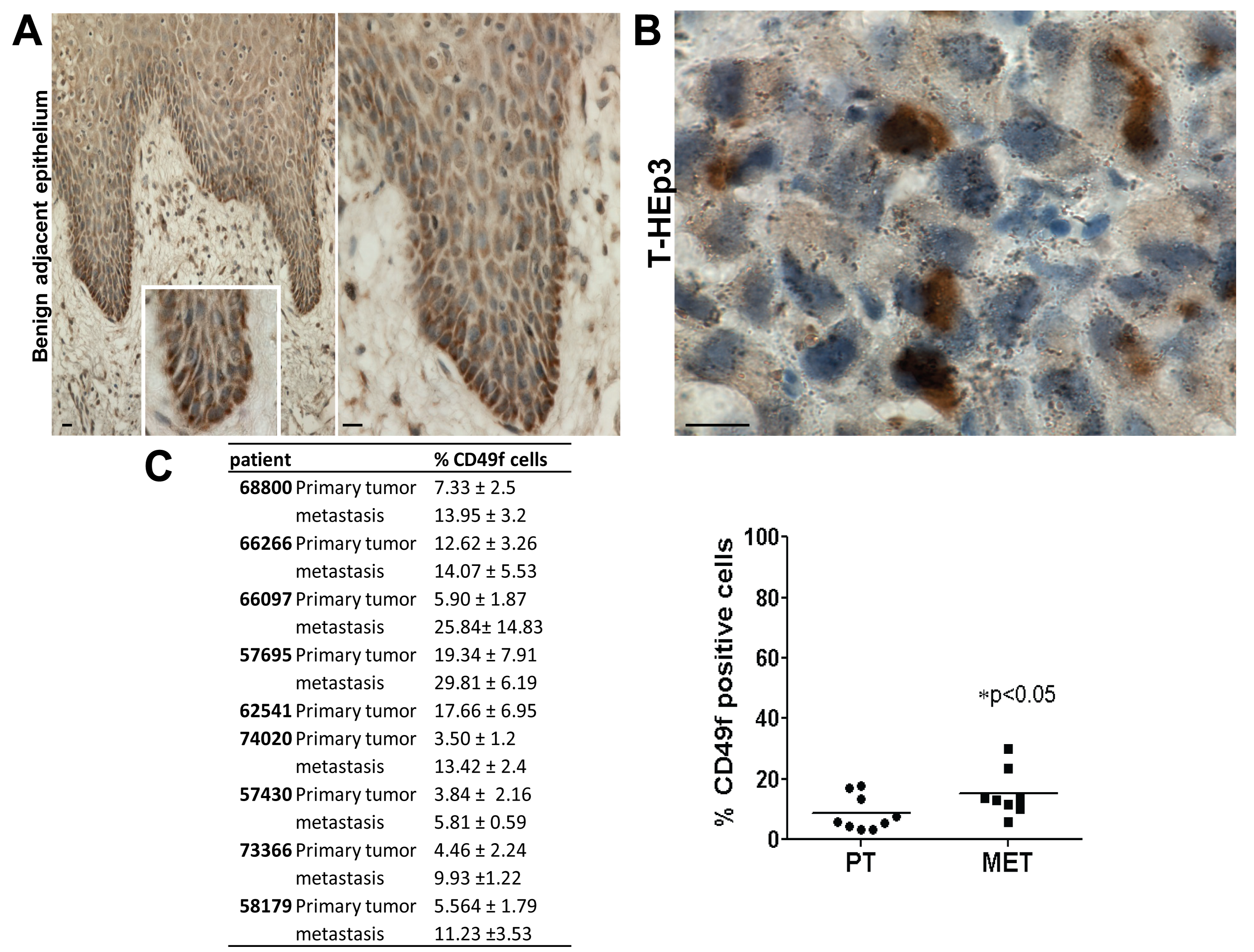

Supplement: Figure S2 — Expression of CD49f in normal epithelium. Representative images of CD49f staining in benign adjacent oral squamous epithelium of patient 68880 (A) and HEp3 tumor (B). Scale bar: 20 µm (left panel) and 40 µm (right panel) in (A). Scale bar: 60 µm (B). (C) Table showing the quantification of the mean percent of CD49f positive cells in the primary tumor and metastasis from each patient. Right panel, graph representing the mean percentage of CD49f positive cells in human oral primary tumors (PT) and lymph node metastasis (met). p-values estimated using Mann-Whitney non-parametric test. (TIF) [file pone.0029974.s002.tif]

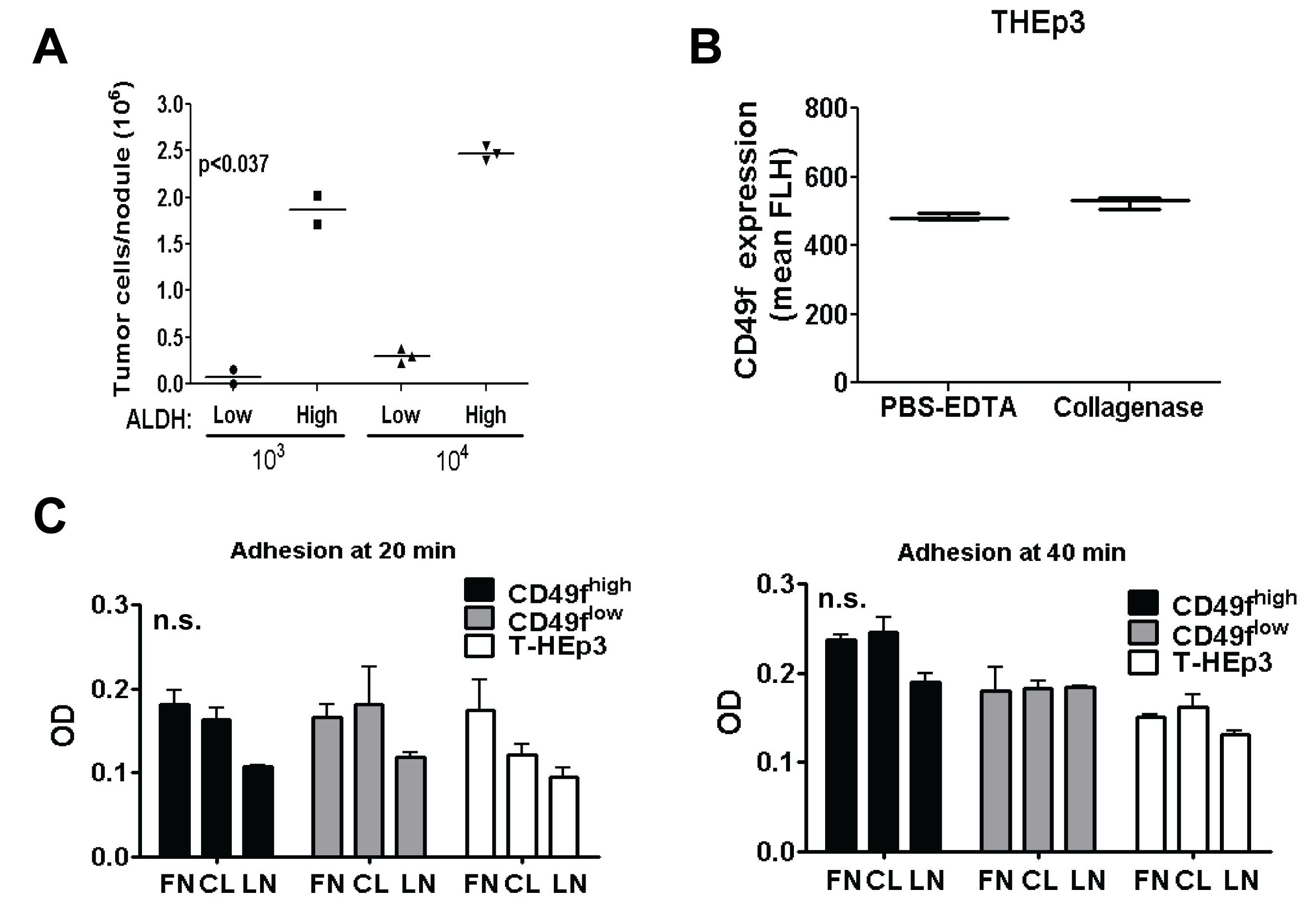

Supplement: Figure S3 — CD49fHigh and CD49fLow cells have similar adhesive potential to the ECM. (A) Quantification of tumor growth after 1 week in vivo of sorted ALDHhigh and ALDHlow populations. Dots represent the number of tumor cells per nodule. p-values estimated using one-way ANOVA followed by the Bonferroni correction with two-tailed P values<0.05 considered significant (B) FACS quantification of CD49f mean fluorescence intensity in HEp3 tumors disaggregated with either collagenase or PBS/EDTA. (C) Adhesion to Fibronectin (FN), collagen (CL) and Laminin (LN) in HEp3, CD49fhigh and CD49flow cells. 25*10∧4 cells per well of 96-well plates, (four wells per experimental point) were inoculated into wells coated with FN 4 mg/ml, CL 4 mg/ml or LN 5 mg/ml incubated at 37 C for 30 min, fixed, and stained with crystal violet (see Methods S1), the dye was extracted, and the absorbance was measured at 570 nm. (TIF) [file pone.0029974.s003.tif]

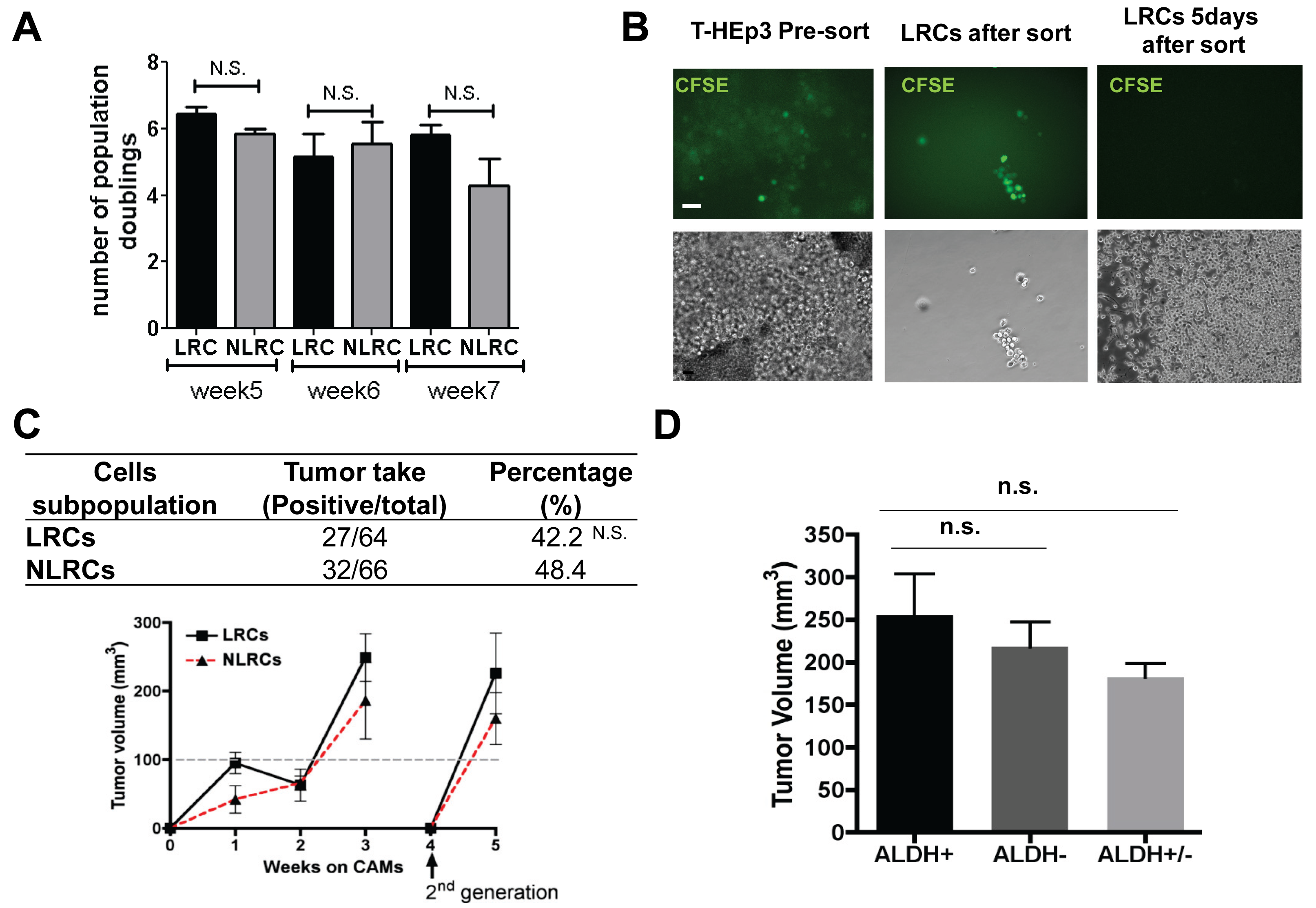

Supplement: Figure S4 — CD49flow/NLRCs can reprogram and regain their tumorigenic capacity. (A) Quantification of the number of population doublings in vivo of LRCs and NLRCs upon serial transplantation for 5–7 weeks. (B) Detection of CFSE in HEp3 cells before sort (left column), immediately after sort (middle column) and 5 days after expansion in vitro (right column). Scale bar 120 µm. (C) Upper table, tumor take for the in vitro expanded progeny of LRCs and NLRCs on CAM after 1 week in vivo. Lower panel graph - LRC and NLRC produced tumors after 3 weeks are able to regenerate again tumors with equal efficiency after 1 week. (D) Quantification of tumor volume produced by the progeny of ALDHhigh (ALDH+) and ALDHlow (ALDH−) and mixed populations (ALDH+/−) after in vitro expansion and reinoculation on CAM. Note that all cells produce tumors of similar size after being expanded in vitro for several weeks. p-values estimated using Mann-Whitney non-parametric test. (TIF) [file pone.0029974.s004.tif]

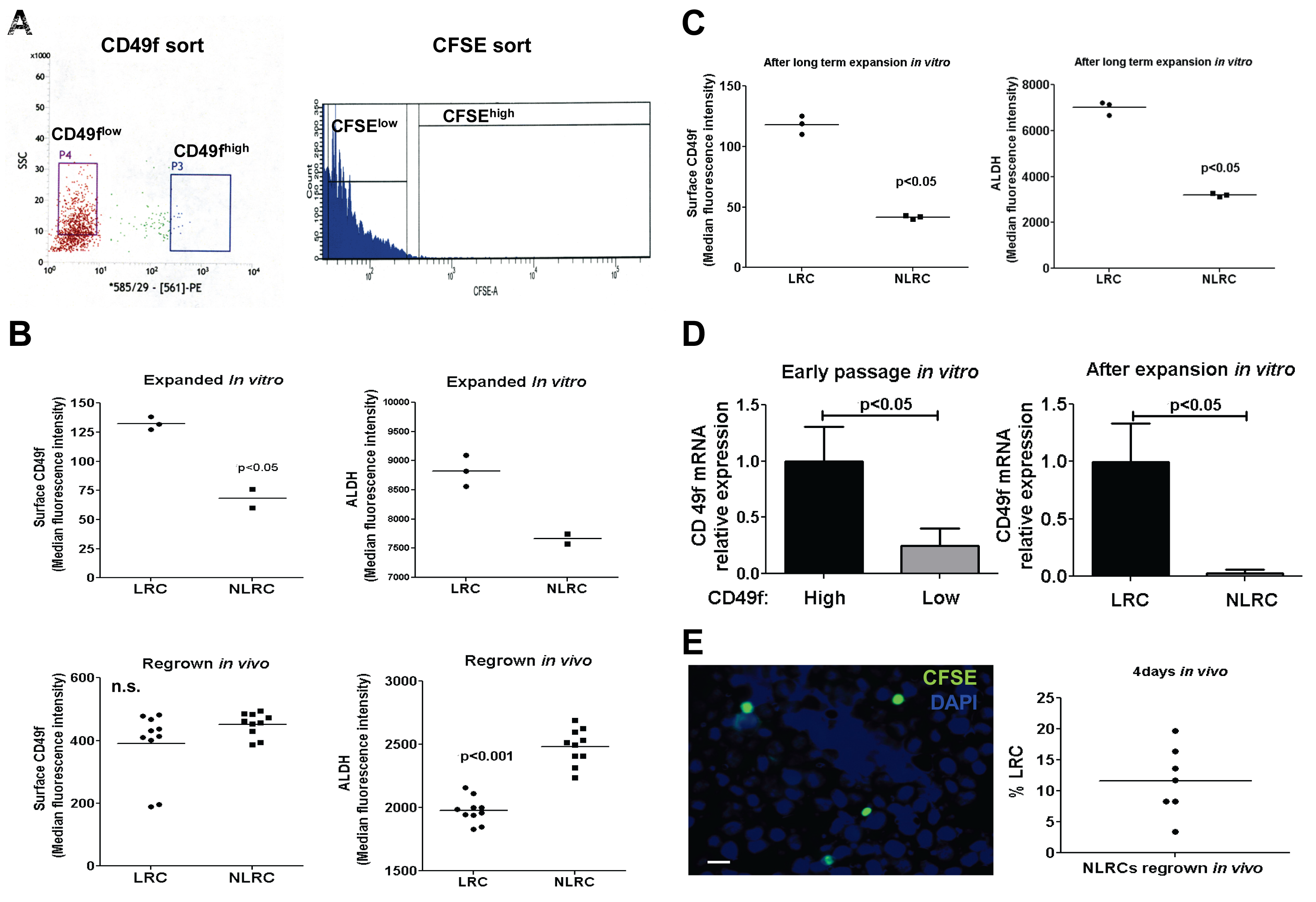

Supplement: Figure S5 — Stability of CD49f expression and ALDH activity in the progeny of HEp3 LRCs and NLRCs in culture. (A) Representative Histograms of purity controls after sort for NLRCs (right panel) or CD49flow cells (left panel). (B) FACS quantification of CD49f (upper and lower left panels) and ALDH1A1 activity (upper and lower right panels) expression in the progeny of LRCS and NLRCs expanded in vitro after sorting (upper panels) from an independent set of tumors from Fig. 5 and after in vitro expanded cells were allowed to form tumors in vivo (lower panels). (C) FACS quantification of CD49f (left panel) and ALDH1A1 (right panel) expression in cells that after sorting were expanded in vitro for more than 100 generations. (D) Quantification of CD49f expression by qPCR in cells that after CD49f sorting were expanded in vitro for 6 passages (left panel) or that after CFSE sorter were expanded in vitro for 100 passages (right panel). (E) The tumor progeny of the NLRCs expanded in culture were labeled with CFSE (20 µM) and inoculated in vivo. NLRC after expansion in vitro regain tumorigenic capacity and are able to generate a LRC population that can be detected by fluorescence microscopy (left panel). Scale bar: 80 µm. Right panel, quantification of the number of CFSE positive cells in 4 days tumors formed by the progeny of the NLRCs. (TIF) [file pone.0029974.s005.tif]

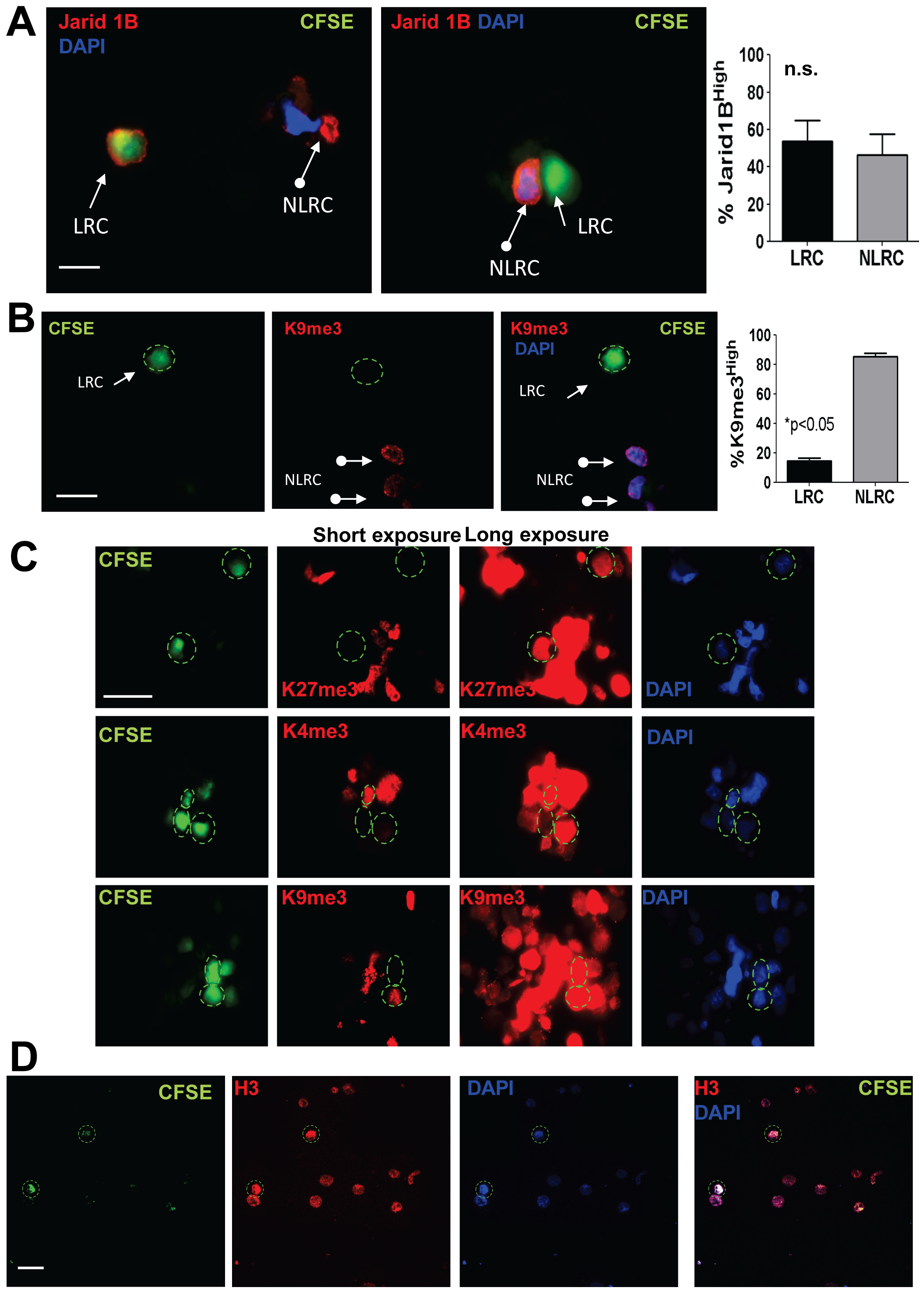

Supplement: Figure S6 — Jarid1B and H3K9me3 expression in HEp3 LRCs and NLRCs. (A–B) Detection of CFSE, JARID1B (A) and H3K9me3 (B) by immunofluorescence in CFSE-labeled HEp3 cells grown in vivo for 4 days. Scale bar: A = 40 µm, B = 60 µm. Quantification of positive cells is shown on the right. Graph shows mean ± SD of three independent tumors. Y axes represent the percentage of cells per tumor. p-values estimated using Mann-Whitney non-parametric test. (C) Detection of CFSE, H3K27me3, H3K9me3, H3K4me3 by immunofluorescence. Here we used a short integration time on the digital camera to reveal the H3PTM high marks (second column). In the third column we used a longer integration time on the digital camera to show that H3-PTM marks are present in those cells that appear negative in the second column. This reveals that it is a degree difference and not a positive vs. negative difference. Scale bar: 80 µm. (D), Detection of total H3 levels in LRCs and NLRCs. Note that all cells are positive for total H3. Scale Bar = 80 µm. (TIF) [file pone.0029974.s006.tif]
