# Supplementary material for: Analysis of Marker-Defined HNSCC Subpopulations Reveals a Dynamic Regulation of Tumor Initiating Properties
Source: PLoS One. 2012 Jan 20;7(1):e29974. doi: 10.1371/journal.pone.0029974 (PMC3262798; doi:10.1371/journal.pone.0029974)
Supplement: Methods S1 — Adhesion assays. Adhesion to Fibronectin (FN), collagen (CL) and Laminin (LN). HEp3, CD49fhigh and CD49flow cells were inoculated into wells coated with FN 4 mg/ml, CL 4 mg/ml or LN 5 mg/ml, incubated at 37 C for 30 min, fixed, and stained with crystal violet, the dye was extracted, and the absorbance was measured at 570 nm. Table of Antibodies. (DOCX) [file pone.0029974.s007.docx]

**MATERIAL AND METHODS S1**

**Adhesion Assays**

Matrix protein or polylysine plates, (96-well) were coated with Fibronectin (FN) 4mg/ml, type I collagen (CL) 4mg/ml or Laminin (LN) 5mg/ml and incubated overnight at 4°C in PBS, and blocked for 1 h at 37°C with 1 mg/ml BSA. Cells were detached with 2 mM EDTA, resuspended in DMEM at 5 × 10^5^/ml, and 25*10^4 cells per well were inoculated. After 20 and 40 min incubation at 37°C, the wells were washed gently, fixed with methanol, stained with 0.5% of crystal violet in water for 10 min, and washed extensively with water. After microscopic inspection, 60 μl of 10% methanol and 5% acetic acid solution was added to each well and, after 10 min, the OD at 570 nm was measured in a microplate reader (Dynatech Laboratory Inc.).

**Table I. Antibodies**

| **Antibody** | **Commercial House** | **Catalogue #** | **technique** |
| --- | --- | --- | --- |
| Human ALDH1 aa. 7-128 | BD | 611194 | IF/FACs |
| Anti-CD49f, clone 4F10, Phycoerythrin conjugated | Cell signaling | CBL458P | IF/FACs |
| Anti-CD49f, clone G0H3 | R&D systems | MAB13501 | Blocking antibody |
| Rat X Hu CD49f | Chemicon International | MAB 1378 | IH |
| Anti CD44 |  |  | FACs |
| Anti-trimethyl-Histone H3 (Lys27) | Millipore | 07-449 | IF/WB |
| Anti-trimethyl-Histone H3 (Lys9), clone 6F12-H4 | millpore | 07-442 | IF |
| EZH2 | Cell signaling | AC22 | IF/WB |
| Jarid 1B monoclonal antibody M02 clone IG10 | Abnova | H00010765-M02 | IF |
| H3K4me3 | Millipore | 04-745 | IF |
| Anti-Histone H3 | Millipore | 06-755 | IF |
| IgG2A Isotype control | R&D systems | maB006 | Isotype control |
| Phospho-PERK (Thr980) (16F8) | Cell signaling | #3179 | IF /WB |
| Alexa fluor 568 goat anti rabbit | invitrogen | A11011 | IF |
